# Predicting the effects of winter water warming in artificial lakes on zooplankton and its environment using combined machine learning models

**DOI:** 10.1038/s41598-022-20604-x

**Published:** 2022-09-27

**Authors:** Marek Kruk, Anna Maria Goździejewska, Piotr Artiemjew

**Affiliations:** 1 Faculty of Mathematics and Computer Science, University of Warmia and Mazury in Olsztyn, Słoneczna 54, 10-710 Olsztyn, Poland; 2 Faculty of Geoengineering, University of Warmia and Mazury, Oczapowskiego 5, 10-719 Olsztyn, Poland

**Keywords:** Biodiversity, Climate-change ecology, Community ecology, Ecological modelling, Freshwater ecology, Biodiversity, Climate-change ecology, Community ecology, Ecological modelling, Freshwater ecology, Ecology, Ecology, Climate sciences, Climate change

## Abstract

This work deals with the consequences of climate warming on aquatic ecosystems. The study determined the effects of increased water temperatures in artificial lakes during winter on predicting changes in the biomass of zooplankton taxa and their environment. We applied an innovative approach to investigate the effects of winter warming on zooplankton and physico-chemical factors. We used a modelling scheme combining hierarchical clustering, eXtreme Gradient Boosting (XGBoost) and SHapley Additive exPlanations (SHAP) algorithms. Under the influence of increased water temperatures in winter, weight- and frequency-dominant Crustacea taxa such as *Daphnia cucullata*, *Cyclops vicinus*, *Cryptocyclops bicolor*, copepodites and nauplii, and the Rotifera: *Polyarthra longiremis*, *Trichocerca pusilla*, *Keratella quadrata*, *Asplanchna priodonta* and *Synchaeta* spp. tend to decrease their biomass. Under the same conditions, Rotifera: *Lecane* spp., *Monommata maculata*, *Testudinella patina*, *Notholca squamula*, *Colurella colurus*, *Trichocerca intermedia* and the protozoan species *Centropyxis acuelata* and *Arcella discoides* with lower size and abundance responded with an increase in biomass. Decreases in chlorophyll a, suspended solids and total nitrogen were predicted due to winter warming. Machine learning ensemble models used in innovative ways can contribute to the research utility of studies on the response of ecological units to environmental change.

## Introduction

Knowledge about the functioning of ecosystems and their components under conditions of climate dynamics is one of the fundamental tasks of environmental science. For aquatic ecosystems and their associated organisms, such key phenomena are atypical fluctuations in air temperature in a given climate zone that trigger adaptation responses by assemblages of aquatic organisms^1^. This is especially true when seasonal changes in water temperature collide with the phenological cycle. In temperate transitional climates, this generally applies to increased water temperatures during the winter months^2^.

Zooplankton are an integral, fundamental component of the trophic network of aquatic ecosystems. Its structure and biomass determine the filtration of phytoplankton, i.e. the purification function of the water body, and the size of the food base for planktivorous fish^3^. Due to their high sensitivity to physico-chemical factors of the aquatic environment, zooplankton communities are used as potential bioindicators^4^ and indicators of water quality^5^, with a particular focus on rotifers^6^. The above studies indicate a high correlation between zooplankton abundance and chlorophyll a concentration and water visibility. We observe a growing interest in the response of zooplankton to thermal changes in the water. The direct response of zooplankton to water thermals affects the regulation of individual metabolism, developmental rate^7^ and relationships with phytoplankton^8^. It influences changes in population size, species composition, phenology and vertical distribution in the water column as an effect of climate warming^9^ and artificial water heating^10^, e.g. rotifers^11^. Indirectly, changes in water temperature can limit phytoplankton as food^12^ and through the activity of ichthyofauna^13^.

Freshwater zooplankton respond to an increase in seasonal mean water temperatures with an increase in density and biomass and a change in species composition, as has been confirmed in many studies^14–18^. A greater proportion of juvenile forms, greater growth of smaller crustaceans^19^ and a reduction in large-bodied zooplankton have been observed^20^. Disturbances in the seasonal succession and recovery of cold-water Rotifera species have also been noted^11^. An earlier onset of the warm season accelerates the development of thermophilic species and often disrupts their life cycle^21^, either in a long-term process^22^. The onset of spring warming determines the rate of stage changes^23^, reproductive success^24^ and food availability^25^. The response of individual species to changes in thermal conditions has a direct influence on the taxonomic structure^24^ and functioning^26^ of zooplankton communities. This structure determines interspecies relationships, including competitive displacement and predation^27^, and affects all trophic levels of the aquatic ecosystem^28^.

The effects of winter thermals on the status of biocenoses during the growing season in inland aquatic ecosystems have not been extensively studied. Research and conclusions tend to focus on the global impacts of climate warming and concern of oceanic and marine zooplankton^29^. Richardson^15^ notes that there is a growing need for research on the effects of thermals on zooplankton in inland waters, although the environmental conditions here are more complex than in the oceans. This topic is addressed in the article describing the effects of the decreasing ice cover of Lake Baikal on plankton trophic networks and phytoplankton bloom cycles. The decreasing ice cover from year to year causes the plankton network to rebuild, to be less intense and shifts the phytoplankton bloom to summer^30^. Changes in the phenology of the phytoplankton and zooplankton of Müggelsee Lake, involving a shift of activity maxima to late spring and summer, have been the subject of advanced modelling in the context of climate warming by Recknagel et al.^31^. Our work attempts to fill a gap in the literature concerning the response of zooplankton to the effect of "flattening" the course of annual average temperatures by increasing the temperature in winter^2^.

This study aims to answer the question of how the perturbation of the natural seasonal variability of lake water thermals in winter affects the prediction of increases or decreases in the biomass of zooplankton taxa. The study was conducted in a complex of morphometrically and hydrologically similar artificial reservoirs draining opencast mines in central Poland. In three of them, winter water temperatures were typical for the climate zone, while in three others winter temperatures were significantly higher. The described situation can serve as a model for generalisations of the response of the zooplankton community of inland waters to the disappearance of long winter periods with low temperatures caused by climate change, as predicted in climate change projections for Europe^32^ and at the global scale^33^.

In this paper, we have made several assumptions that are necessary to better understand the phenomena at the aquatic ecosystem and zooplankton assemblage level. First, we have treated the biomass of zooplankton species as a multidimensional system of interactions between species in the biocenosis^34^. Such properties are the basis of the boosting modelling technique^35^, which is based on random selection of interactions between factors and uses boosting of single variables (so-called weak learners) to obtain a model with the highest accuracy, with the maximum number of so-called strong learners. Boosting mimics the interactions between species in a biocoenosis that counteract the tendency to lose cohesion and survival^36^. The use of the algorithm eXtremeGradientBoosting (XGBoost) allowed the prediction of the modelling results and the evaluation of their accuracy measures. We assumed that high values of this measure confirm the accepted thermal classification of the studied lakes and its impact on zooplankton species. Given the increasing use of machine learning in ecological research, there is a need to use appropriate tools to visualise and interpret the obtained model prediction results so that they reflect the ecological reality of the modelling as fully as possible^37^. To this end, we added modelling with SHapley Additive exPlanations (SHAP) to the boosting model. This algorithm was used to calculate and visualise individualised interactions between environmental variables in the analysis of feature selection for stream water quality monitoring^38^, the prediction of microbiological water quality of coastal waters^39^ and the analysis of the sensitivity of environmental parameters of lagoon waters to annual weather changes^40^. In our study SHAP modelling was used to assess the effects of winter warming based on the interactions between the biomass of zooplankton species in the two thermal artificial lake types.

We have assumed that there is some form of survival game between zooplankton taxa, where populations of a species co-evolve as trophic or competitive guilds^41^. In a broader perspective, the interactions between biotic and abiotic environmental factors can also be considered as a kind of adaptive game, e.g. in a universal sense^42^ and in the context of environmental change^43^. A formal description of the functioning of such a system can be sought in mathematical game theory, or more precisely in corporate game theory. Following this assumption, to model the response of the biomass of the zooplankton taxa of the studied reservoirs, we used the SHAP modelling developed on the basis of the Shapley value theory^44^ and the SHAP algorithm^45^. This modelling, based on a binary classification, can be used to show the importance of features in a classification model built from the bottom up from the values of individual cases or observations^46^. It allows us to indicate the direction and strength of the response of the taxon biomass variable to the assumed effect, i.e. increased winter water temperatures in our study. The introductory modelling to obtain predictions in the model SHAP is the XGBoost algorithm with the boosting technique described above, which ensures high interactivity of the variables included in the SHAP modelling.

In this study, we sought to investigate the effectiveness of SHAP modelling for numerous zooplankton taxa, including species with low abundance that can nevertheless serve as indicators of environmental change. To more comprehensively investigate interactions in a community that consists of several dozen taxa and is highly diverse in terms of biomass and abundance, we have proposed an alternative procedure that involves the inclusion of a hierarchical clustering method in the modelling sequence^47^. This is the first time in the ecological literature that a clustering method and Shapley value-based modelling have been combined to provide the most realistic predictions of how an assemblage of organisms will behave in the face of environmental change. Our work is the first in the zooplankton literature to introduce a methodological approach based on machine learning of clusters and classification explanatory modelling.

This study aims to demonstrate the possible influence of 'winter warming' on the biomass response of zooplankton species in small artificial flow-through lakes through innovative modelling with machine learning. In this paper, we also verify the thesis that prediction methods based on self-learning systems are needed to reveal structural and functional changes in communities of aquatic organisms due to environmental changes. By modelling SHAP we show how useful it is to analyse a broader ecosystem context to explain phenomena at the level of interactions between species in the biocoenosis.

## Results

### Water thermic features of artificial Cold and Warm Lakes

**Figure 1:**
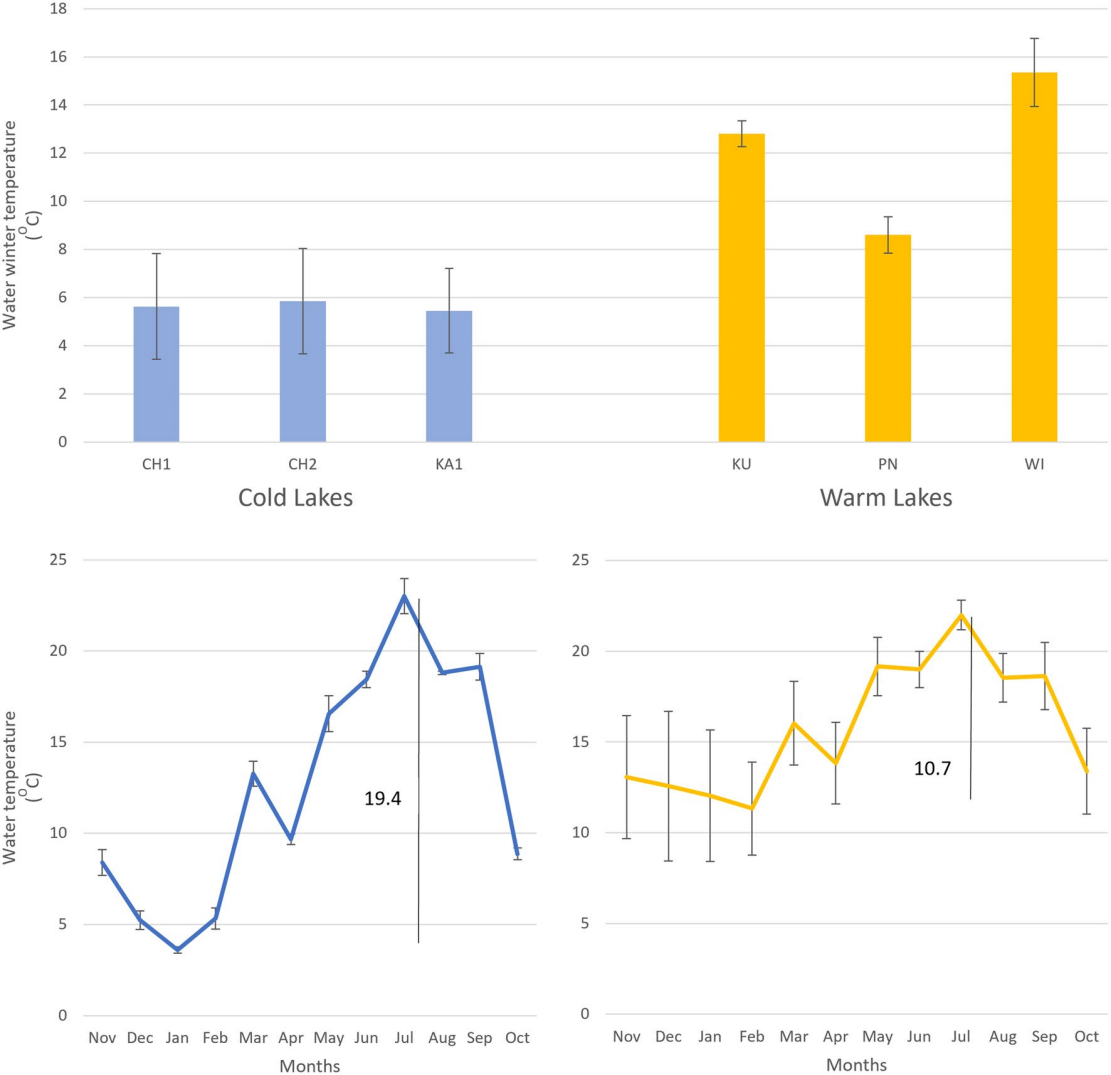
Thermal properties of Cold and Warm Lakes. Upper graph: Water temperatures in three Cold and Warm Lakes during the winter months of December–February. Lower plots: Dynamics of water temperature during the year from the lowest temperature in winter (January/February) to the highest in summer (July). The amplitudes of the annual temperature are marked with vertical lines.

The thermal conditions of the two reservoir classes, Cold Lakes and Warm Lakes, showed two very distinct differences. Firstly, average winter temperatures (November-February) were on average 3 to even 9 °C higher in the Warm Lakes. Winter temperatures in the Cold Lakes did not exceed 6 °C on average, while in the Warm Lakes they varied between about 8.5 and 15 °C (Fig. 1, upper diagram). A significant difference between these types of reservoirs existed in the annual course of water temperatures. The course of water temperature in the Cold Lakes is more dynamic than in the Warm Lakes. The amplitude between the coldest winter month and the warmest summer month was 19.4 °C on average in the Cold Lakes, while it was almost twice as high in the Warm Lakes at 10.7 °C (Fig. 1, lower graph).

### Zooplankton taxa diversification among lake thermic classes

The differentiation of zooplankton taxa between Warm and Cold Lakes, and the identification of those whose biomass increased under the influence of warmer water in winter is shown by comparing the average biomass in both thermal types of reservoirs. Of the 76 species studied (Table S1), 28 showed significant differences in biomass, with the vast majority, 24 taxa, having higher biomass in the Cold Lakes and only 4 in the Warm Lakes (Table S1). In the Warm Lakes, only 3 Rotifera taxa had higher biomass: Cephalodella spp., Colurella colurus and Euchlanis spp. and one protozoan species Arcella discoides. The abundance of these species was 3 to 7 times higher in the reservoirs with warmer water (Table S1).

Cold and Warm Lakes also differed in terms of species richness. Of 76 taxa in all reservoirs (with abundances greater than 1%), 62 were identified in the Cold Lakes and 69 in the Warm Lakes. We observed more Rotifera (47) and Cladocera (8) species in the warmer reservoirs than in the colder reservoirs (39 and 6). In contrast, significantly more Copepoda taxa were found in the Cold Lakes (12) than in the second type of reservoirs (8) (calculated from Table S1).

### Clustering modelling of zooplankton taxa

**Figure 2:**
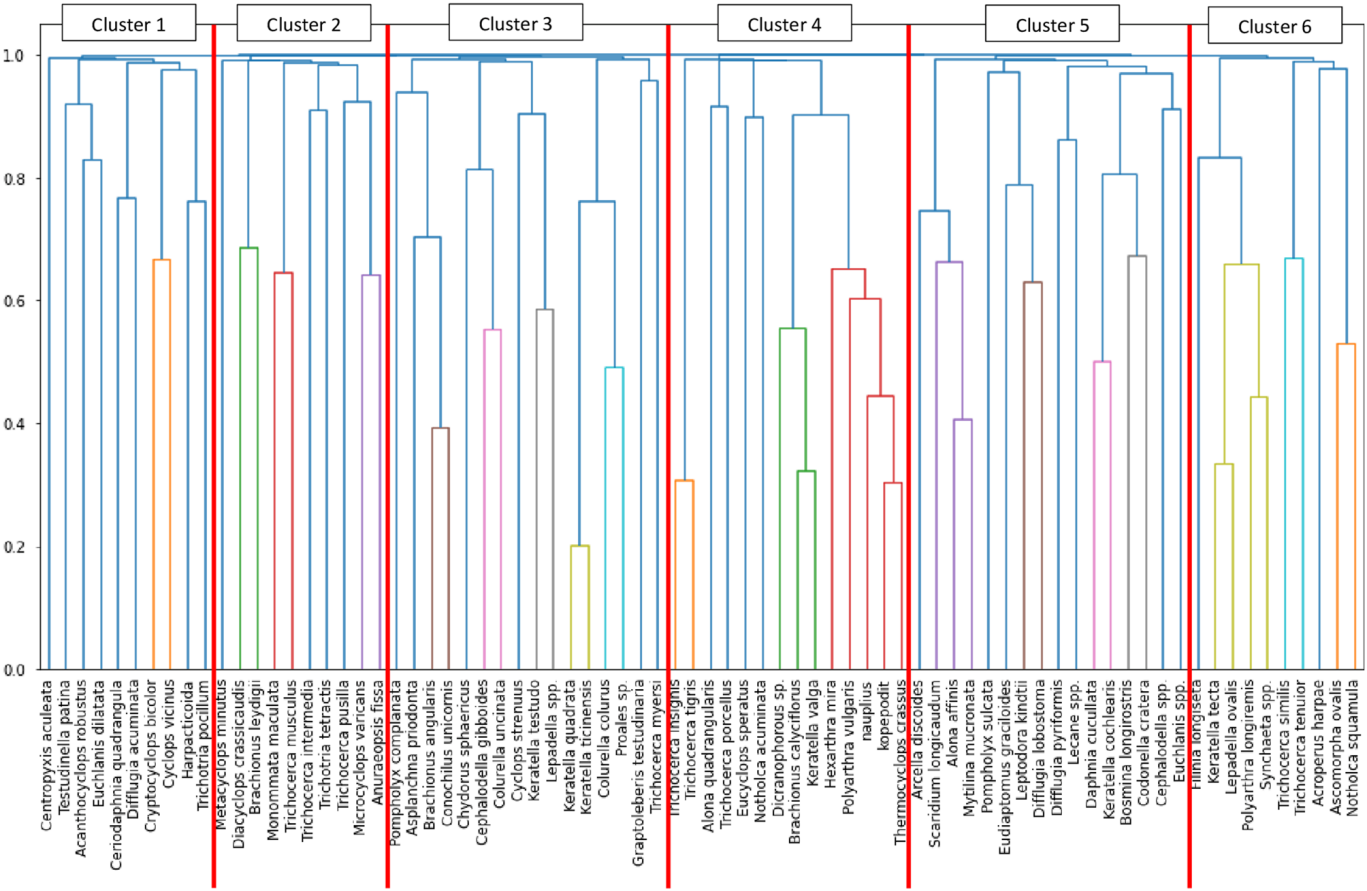
The dendrogram of the hierarchical clustering of the zooplankton taxa of the Cold and Warm Lakes. The threshold for 6 clusters is 0.998. Results of the dendrogram metrics: Silhouette Score: 0.425, Calinski-Harabasz Score: 129.2807 and Davies Bouldin Score: 0.7925. A sequence of 9 colours, except deep blue, indicates the smaller clusters with a distance threshold of 70% of the final merge (Python library Scipy documentation).

The set of 76 zooplankton taxa was divided into 6 groups ranging from 10 (clusters 1, 2 and 6) to 16 (clusters 3 and 5) taxa. This subdivision was achieved by a threshold of 0.998. The dendrogram is characterised by a Davies-Baouldin index of 0.7925, which links internal cluster cohesion with inter-cluster cohesion (Fig. 2).

The different clusters were characterised by a different taxonomic composition in terms of the presence of species from the three zooplankton groups Crustacea, Rotifera and Protozoa. The cluster with the highest relative proportion of Crustacea was Cluster 1 (5 species in relation to 3 Rotifera and 2 Protozoa). Cluster 5 was the most balanced in terms of composition (5 Crustacea taxa, 7 Rotifera, 4 Protozoa). In the other clusters, the Rotifera species predominated on Crustacea, especially in clusters 3 and 6. At the same time, protozoan species were absent (Fig. 2).

### Prediction the warming effect on zooplankton taxa – SHAP cluster models

**Figure 3:**
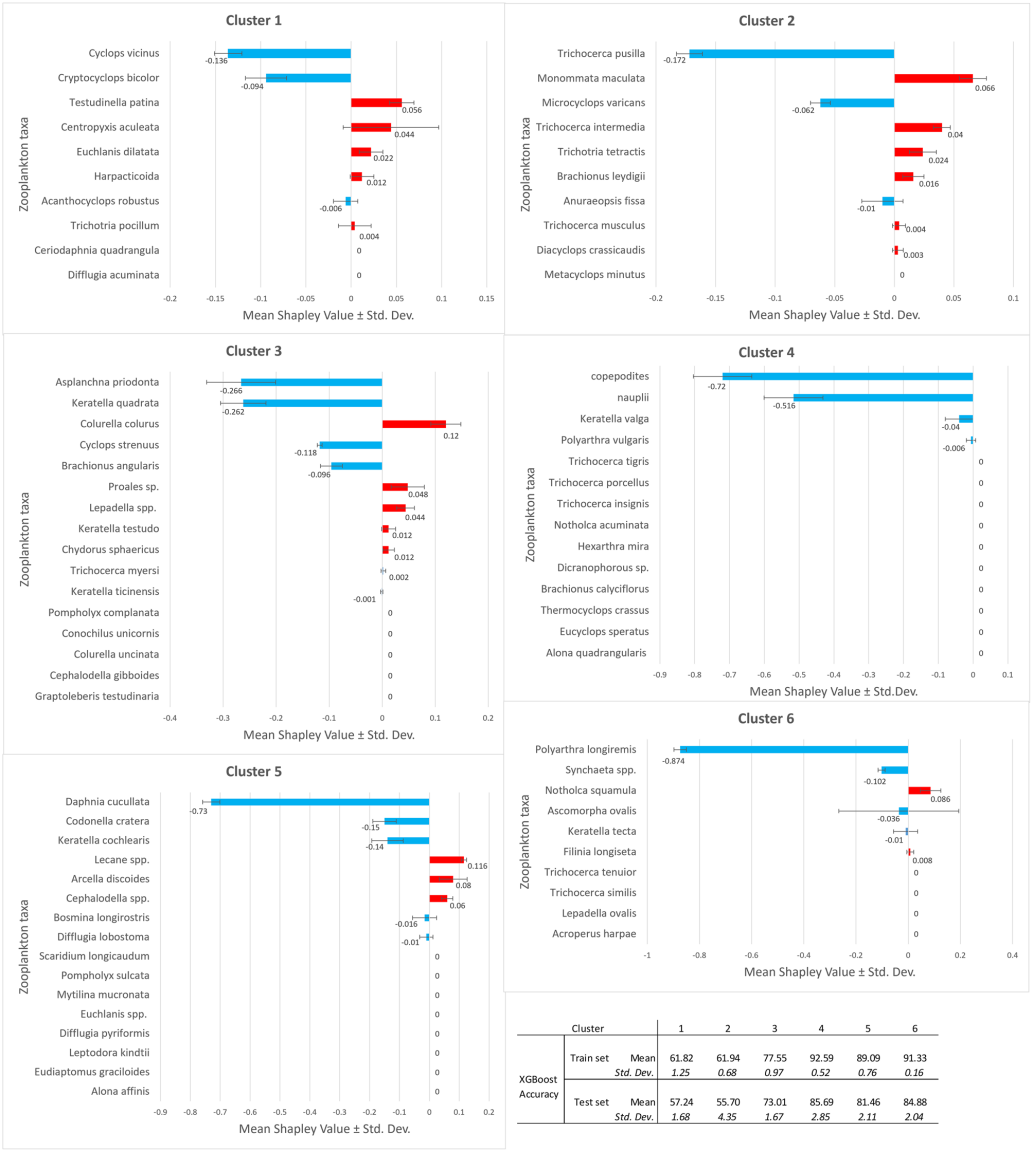
The SHAP has created six synthesis models for the response of zooplankton taxa to winter water warming. The models were built based on the cluster modelling results (Fig. 2). Each of the six SHAP cluster models shows the mean and standard deviation of five randomly selected individual SHAP modelling results. The values of the observations are shown: red bars and a positive Shapley value indicate higher biomass due to winter water warming, blue bars and a negative Shapley value indicate lower biomass. The table shows the mean and standard deviation of the five XGBoost models for each of the six SHAP cluster models. The distribution results of the individual SHAP models can be found in the Supplementary Information (Fig. S1).

Clustering of the zooplankton community in Cold and Warm Lakes led to a different prediction of the response of taxa to higher water temperatures in the winter months (Fig. 2). A Shapley value different from 0 was achieved by 46 taxa. The number of taxa with a positive Shapley value with a significant response (Shapley value > 0.04) occurred in 11 taxa. The biomass of *Colurella colurus*, Lecane spp., *Notholca squamula*, *Arcella discoides*, *Monommata maculata*, *Cephalodella* spp., *Testudinella patina*, *Proales* spp., *Centropyxis acuelata*, *Lepadella* spp. and *Trichocerca intermedia* increased as a result of warming of the waters in winter (Fig. 3). Smaller changes were observed in species whose biomass decreased under the influence of warmer winter waters. Here, the lowest negative Shapley values were obtained by *Polyarthra longiremis*, *Daphnia cucculata*, copepodites and nauplii. Below a Shapley value of − 0.100 were taxa such as *Asplanchna priodonta*, *Keratella quadrata*, *Trichocerca pusilla*, *Codonella cratera*, *Keratella cochlearis*, *Cyclops vicinius*, *C. stenuus* and *Synchaeta* spp. (Fig. 3).

In all six clusters, the highest absolute Shapley values were obtained by species for which the model predicted a decrease in biomass. In clusters 1, 2 and 3, the biomass of more species increased than decreased under the influence of winter water warming. These are also the clusters with the greatest number of taxa responding to these changes in water thermals. In contrast, cluster 4 is the worst group in terms of the number of responding taxa (only 4) and this response, i.e. only a decrease in biomass (Fig. 3).

### Prediction of warming effect on lake physicochemical parameters

Several physico-chemical parameters were found to be both an advantage for the Warm Lakes over the Cold Lakes and vice versa. Significantly higher mean values for winter water temperature and Secchi disc visibility were measured in the Warm Lakes, while mean values for oxygen, chlorophyll a, nitrate nitrogen, total nitrogen and suspended mineral matter were higher in the Cold Lakes (Table S2). At the same time, the mean values for annual water temperature, phosphate and total phosphorus concentration, ammonium nitrogen and suspended organic matter concentration showed no significant differences between the artificial lake classes studied (Table S2).

**Figure 4:**
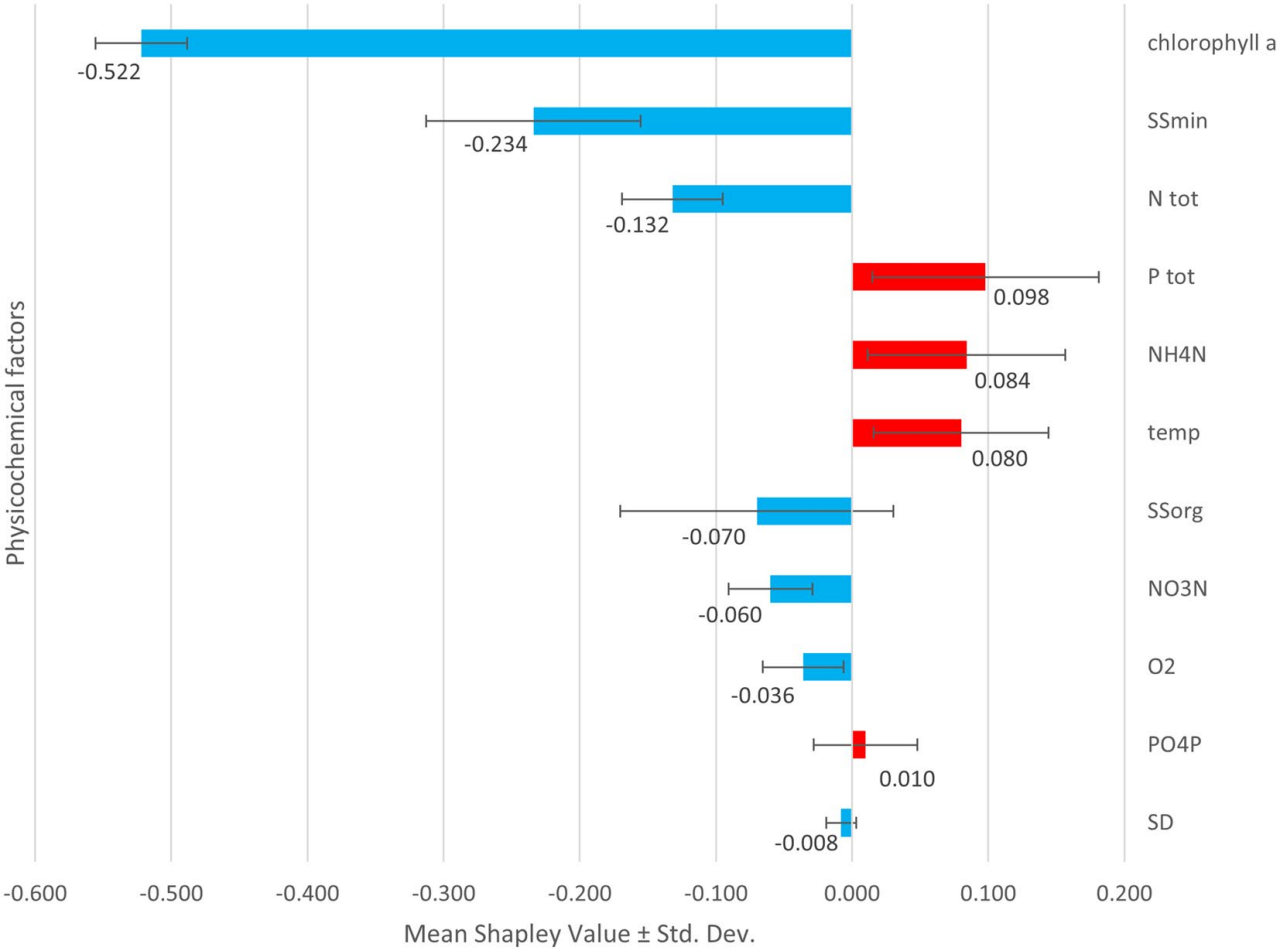
The importance of physico-chemical factors during the study period as an effect of water warming during winter in the classification XGBoost-SHAP modelling. Mean and standard deviation of Shapley value of 5 running models (Fig. S2). The abbreviations are explained in the subsection Methods, Sampling and laboratory works and in the Supplementary Information, Table S2.

Figure 4 shows the influence of winter warming of reservoir water on the response of physicochemical factors of these waters. Chlorophyll a was found to respond most strongly to the winter warming anomaly, and its decrease plays the most important role in the adopted classification model with Cold and Warm Lakes. Similarly, the factors mineral suspended solids and total nitrogen have a negative but smaller influence on the model. Parameters that responded positively to the increase in winter water temperature were total phosphorus, ammonium nitrogen and water temperature (Fig. 4).

## Discussion

### Modelling approach

Modelling of SHAP was chosen in this study to realistically evaluate predictions of zooplankton changes associated with changes in water thermals in artificial reservoirs. With a pre-applied boosting technique, it relies on multiplicative interactions between taxon biomass and model learning^45^. The interpretive power of the XGBoost–SHAP model ensemble used to assess the importance of ecological drivers of environmental change, including climate, far exceeds the conclusions that can be drawn from, for example, an analysis of variance. If we compare the significant differentiation in the biomass of zooplankton species between the Cold and Warm lakes of the Kruskal–Wallis test with the Shapley values, the differences become visible. Using the Kruskal–Wallis test (P < 0.05), we have 24 species with a higher biomass in Cold lakes and 4 species in Warm lakes (Table S1), while the Shapley value > 0.02 indicates a lower biomass due to winter warming for only 17 species and a higher biomass due to winter warming for as many as 13 species.

Modelling based on interactions as a game between environmental factors mimics actual processes both in the ecosystem and in nature in general^42^. In living organisms, it is primarily an adaptation to a changing environment as a feedback system^43^. The principle of species survival and gene transfer determine the rules of their "game in the ecosystem"^48^, in the broader sense of evolution^49^ and with reference to computational biology^50^. Modelling the ecosystem with the boosting technique is more effective than using generalised additive models (GAM)^51^. Changes in plankton community phenology related to climate warming have been modelled by Recknagel et al.^31^ using the hybrid evolutionary algorithm^52^. One technique to achieve multiple interactions between phytoplankton and zooplankton groups was bootstrapping, followed by modelling based on evolutionary algorithms^31^. The authors conclude that future studies should expand the ensemble of inference models to include endogenous factors that reflect competition and predation between plankton groups.

The reality of interactions between populations of species in a biocoenosis includes not only the largest or most abundant species, but the entire taxonomic spectrum of the community. Smaller species with lower biomass can be an important component overall in an interactive, multidimensional^34^ game for food resources by forming direct and indirect relationships with the dominant species and with each other^53^. They can also be sensitive indicators of environmental change^54^. To delve deeper into the structure of these interactions, a tool is needed that increases the "resolution" of vision and the ability to analyse and predict their responses. First of all, it should be assumed that the interactions in the zooplankton community can be classified into a certain hierarchical structure and links should be sought between some stronger taxa and others that are weaker. The solution we applied in this case was the hierarchical clustering technique^55^.

The clustering method has been used in zooplankton studies for several years. This has usually involved looking for geographical similarities in the characteristics of taxa. For example, this type of modelling was used to determine the similarity of 50 species of zooplankton biomass in the Chukchi Sea^56^. The cluster dendrogram was applied to functional traits by Pomerleau et al.^57^ using an average linkage clustering of 42 zooplankton species. Hierarchical clustering, based on the strength of links between zooplankton taxa using Euclidean distance and measured cophenetic correlation, has proven useful in modelling the effects of environmental change on zooplankton functional diversity. For example, it has been used to analyse relationships between oceanic and coastal Copepoda species on the Brazilian coast^58^. It has also been used to determine the effects of climate change on zooplankton diversification in the Mediterranean^59^. We report these studies to show that the application of advanced hierarchical clustering to zooplankton analyses can serve several research purposes. They have focused primarily on marine and oceanic ecosystems, and to a much lesser extent on inland waters. This work aims to reduce this discrepancy.

The modelling sequence proposed in this paper, including clustering as an innovative first step, is a continuation of earlier considerations on the application of the SHAP model to predict the sensitivity of the abiotic and biological environmental factors of the Vistula Lagoon to the dynamics of meteorological conditions in subsequent years^40^. In the cited study we used a wide range of ecological factors as variables, including the biomass of zooplankton species. In this study, we focused only on this group of organisms. Given the large number of zooplankton species (variables in the model), we used an innovative method of clustering to obtain detailed information about their response to changes in water temperature in winter. When comparing our work with similar studies using SHAP^38,39^ modelling, it should be noted that we applied the final averaging of a pool of five models with the Shapley value results (Figs. 3 and 4). We believe that this increases the credibility of the conclusions obtained, especially when we are dealing with a relatively small size and diverse data. It should be emphasised that the application of individualised modelling based on Shapley theory is still in its infancy in environmental science and this method, which has a high explanatory value, is likely to be improved in the near future.

### Winter warming effect on the zooplankton

The choice of small artificial lakes, which serve as settling basins for mining wastewater, for studies on the influence of climatic phenomena on aquatic organisms has important advantages over natural objects. The system for draining an opencast lignite mine created the unique situation of creating aquatic ecosystems that differ in the thermal conditions of the water supplied to the basins from different depths of the opencast mine. The operation of these thermal lake types for about a quarter of a century created separate environmental systems that differed in several physicochemical and biological characteristics due to the thermal differences. We could assume that the three reservoirs fed by warm water (Warm Lakes) are a projection of the conditions we will find years later in lakes with cooler water (Cold Lakes).

The typical conclusion regarding thermal effects in the form of increased water temperature on the biomass of aquatic plankton organisms is an increase. This is due to the ectothermic metabolism of phytoplankton as primary production in aquatic ecosystems^60^. As a result, algal blooms, including toxic cyanobacteria, occur under favourable light conditions and nutrient supply^61^. Zooplankton biomass generally increases when phytoplankton are consumed^62^.

Given the global response of the zooplankton community to climate warming outlined above, the conclusions drawn from this study of small inland waters may well be conflicting. The prediction of the biomass of zooplankton taxa indicates a predominant biomass loss of the dominant zooplankton species, while the analysis of the response of environmental factors reveals a decline in chlorophyll a concentration as the most important environmental factor under conditions of high winter water temperatures in the studied reservoirs.

Let us take a closer look at the changes and seasonal dynamics of the temperature course in the water reservoirs, the situation of the plankton organisms, the changes in their biomass and diversity. The comparison of the data on the course of water temperatures in the Cold and Warm Lakes, the prediction of the biomass response of the zooplankton taxa and the values of the physico-chemical factors of the water in these reservoirs due to the winter warming of the water allows us to propose the following stages of ecosystem change.

1. Increased water temperatures in winter are neither an episodic phenomenon nor an experiment, but the result of a long-term inflow of thermally quite stable groundwater, including from geothermal sources^63^, which is warmer in winter than the water of surface or shallow subsurface origin.
2. In the growing season outside winter, the course of water temperatures in "heated" reservoirs is similar (somewhat flatter) than in colder reservoirs. To reach summer temperatures, i.e. above 20 °C, the water in the colder lakes had to warm up by 19.4 °C from January to summer, while in the warmer lakes it only rose by 10.7 °C in the same period (Fig. 1).
3. If the water temperature in the Cold Lakes rises with high dynamics after winter, i.e. the heat energy is absorbed by the water surface, there may be a faster and more abundant development (bloom) of phytoplankton. In contrast, phytoplankton production may decrease when the water temperature curve is flattened (less energy from the temperature difference), as is the case in Warm Lakes. In such a case, colonisation of the water surface by macrophytes (Fig. S3) and further displacement of phytoplankton by competition (Fig. 4) become likely. Other effects include a reduction in the concentration of suspended solids (SSmin, SSorg) and total nitrogen (Ntot) in the water (Fig. 4). Nitrogen compounds could be intensively taken up by the macrophytes.
4. The decline in phytoplankton production is followed by a decline in the growth of its consumers, especially the most fastidious and biomass-rich Cladocera such as *Daphnia cucullata*^64^ and the effective philtre-feeding larvae of copepodites and nauplii^65^, either after mining activity^66^. Rotifers indicating increased trophic water conditions, such as *Trichocerca pusilla*, *Keratella quadrata* and *Anuraeopsis fissa*^6^, as well as the actively foraging (raptorial) *Polyarthra longiremis*, *Asplanchna priodonta*, *Synchaeta* spp.^67^ (Fig. 3).
5. At the same time, a positive response to environmental changes caused by higher water temperatures in winter can be observed in the reservoirs studied. The biomass was increased by small, i.e. less food-requiring Rotifera species: *Lecane* spp., *Monommata maculata*, *Testudinella patina*, *Colurella colurus*, *Notholca squamula*, *Trichocerca intermedia*, *Proales* spp., *Lepadella* spp. and *Cephadella* spp.^68^ as well as protozoan species: *Centropyxis aculeata*, and *Arcella discoides* (Fig. 3).

In the scenario presented, a fundamental structural change of the ecosystem at the level of primary production takes place in key stage 3: the replacement of phytoplankton by macrophytes. If the cause of these changes is related to a warming of the water temperature in winter, there are two explanations for this process. The first is the energy effect mentioned above. Phytoplankton production doubles with every 10 degree increase in water temperature^69^. Under conditions with higher water temperatures in winter and a "flattening" to about 10 degrees difference in water temperature during the year in the Warm Lakes, algal growth would be twice as low as under natural conditions with an annual water temperature amplitude of about 20 degrees in the Cold Lakes (Fig. 1). Lower production and changes in phytoplankton structure as well as a weakening of algal blooms were observed in the waters of Lake Baikal during ice-free winters, i.e. warmer than during typical ice cover in this climate^30^.

The observation that is probably related to the phenomenon described and has an impact on the reduction of phytoplankton biomass could be an increase in algal feeding by zooplankton (e.g. copepods) due to an increase in their metabolic rate as water temperature increases^70^. This factor, indicated by global models of changes in phytoplankton biomass due to climate warming and constant oceanic waters, could be the cause of the inhibition of growth of this important component of oceanic primary production^71^. Another reason for the inhibition of phytoplankton (e.g. cyanobacteria) growth could be a disturbance of stoichiometric relationships in the form of limited availability of phosphorus relative to nitrogen for cyanobacteria^72^ in the nutrient-phytoplankton feedback loops^73^. In the context of this study, we do not have access to hydrochemical data from an early stage of thermal alteration in the reservoirs.

In warmed reservoirs, we found a decrease in the biomass of most copepod taxa, both those characteristic of astatic habitats and small macrophyte-rich reservoirs (*Microcyclops varicans*, *Cryptocyclops bicolor*) and eurytopic species (*Cyclops vicinus*, *C. strenuus*). Together with the above-mentioned features of "flattened" thermal variability and consistently low food availability in warm reservoirs, the copepod assemblage response was the result of long-term ecosystem disturbance. For example, the lack of pronounced seasonal fluctuations in water temperature in warm reservoirs may have disrupted the developmental cycle (lack of diapause) of copepods, which according to Rybak and Błędzki^64^ may lead to reduced adult body size and biomass, phenological changes^24^ and life history traits^23^. The probable need for different food sources in a situation of planktonic algal scarcity could promote the direct development of the adult stage of Copepoda bypassing diapause, according to Santer and Hansen^74^. Our results are consistent with the conclusions of the two experiments mentioned above that a decrease in copepod biomass due to warm winters indicates a functional adaptation. The results of modelling the plankton community of Lake Müggelsee under the influence of warming and eutrophication provided further evidence for a clear phenological asynchrony of cyanobacteria and cladocera^31^.

Lower concentrations of suspended mineral matter in warm reservoirs were primarily the result of low enrichment of the deep water feeding the reservoirs, but also a decrease in suspended matter in year-round macrophyte habitats. Bacteriophagous rotifers and detritivores—*Keratella tecta*, *Brachionus angularis*, *K cochlearis*—responded to low suspended sediment concentrations with a decrease in biomass. Thanks to their adaptation to different food sources, in situations where planktonic algae are scarce or the species composition is insufficient (e.g. dominance of toxic cyanobacteria), these species use alternative sources such as bacterial "films" deposited on solid substrates (e.g. suspension)^75^ or dead organic material^76^. The density of *Brachionus angularis* and *Keratella cochlearis* was lower in the lake heated by power plant water than in lakes with natural thermals. At the same time, an increased density of Rotifera was only found in Psammon and epiphytic communities^11^.

### Final remarks and conclusions

This work represents a novel numerical approach to study the behaviour of zooplankton in the face of environmental change. Most importantly, we would not have obtained a complete quantitative picture of changes in the biomass of zooplankton taxa under the influence of a perturbation of the annual thermal cycle without the incorporation of advanced modelling based on machine learning algorithms that mimic natural biocenotic processes. As an innovation in the literature, we proposed to include a hierarchical cluster analysis in the XGBoost- SHAP modelling pathway to avoid an oversimplified model of changes in zooplankton assemblage based only on the large number of taxa. This procedure made it possible to represent the response to thermal changes in the water also based on the weight of smaller and less numerous Rotifera and Protozoa species. It definitely increased the information and interpretation value of the SHAP model predictions. It seems that the proposed procedure can be useful to refine the modelling results not only of taxonomic databases, but also of other classification tasks with numerous attributes that differ significantly in their value.

In conclusion, the methodology applied, based on innovative data science tools, has made it possible to obtain a detailed picture of the structural transformation of the zooplankton community in artificial lakes exposed to long-term thermal changes during the winter period. Under the influence of increased water temperatures in winter, the dominant Crustacea taxa in terms of weight and abundance, such as *Daphnia cucullata*, copepodites and nauplii, as well as the widespread Rotifera species *Polyarthra longiremis*, tended to reduce their biomass, i.e. to retreat. In contrast, many Rotifera and protozoan species of smaller size and abundance respond by increasing biomass under the same conditions. The use of SHAP models has also clarified the environmental context of these changes. Indeed, it was found that disturbed water temperatures in winter reduce chlorophyll a concentrations, i.e. reduce phytoplankton, the main food of zooplankton animals. The authors are convinced that the use of combined XGBoost- SHAP modelling in conjunction with hierarchical clustering can contribute to new analyses of the response of species, biocenoses and entire ecosystems to contemporary environmental changes.

## Methods

### Study area

The work concerned six artificial lakes (CH1, CH2, KA1, KU, PN, WI) near the Bełchatów open-cast lignite mine in central Poland (51°24′43.6 "N; 19°26′32.9 "E). The reservoirs serve as sedimentation basins for the drainage network of the Bełchatów and Szczerców opencast lignite mines^77^. Their main function is to reduce suspended sediment through sedimentation, but they are also used for recreational fishing^78^. The reservoirs are flow-through (residence time approx. 16 h), have a similar structure, shape and surface area (7.1–8.2 ha) and depth (1.7–2.7 m)^79^. The water feeding the reservoirs comes from different depths of the outcrops and is characterised by different physical and chemical parameters^77^.

The water feeding the individual reservoirs is characterised by different thermals, as it comes from different depths of the outcrops. Reservoirs CH1, CH2 and KA1 are filled with surface water that collects at temperatures close to those of the air due to precipitation, snowmelt and ground swelling. The water feeding the reservoirs KU, PN and WI comes from deep drainage, from 40 to 350 m depth and has a temperature > of 22 °C, which corresponds to thermal water.

For this study, the reservoirs were divided into two thermal classes: those with lower water temperature in winter (Cold Lakes: CH1, CH2, KA1) and those with much higher winter temperature (Warm Lakes: KU, PN, WI). Figure S3 shows the characteristic differences in water surface cover by vascular vegetation between a typical Cold Lake (CH1 and CH2) and Warm Lake (WI) reservoir. The surface of the CH reservoir was free of higher vegetation, while the WI reservoir was partially covered by floating Nuphar lutea plants. These are visible as light green patches on the satellite image (Fig. S3, photo 4).

### Sampling and laboratory works

Zooplankton were sampled every four weeks in each month of 2014, from March to October 2015 and from June to September 2016. Three sampling sites were set up in each reservoir—in the middle, in the coastal zone and near the philtre zone. Samples were collected using a 5-L Patalas trap from a depth of about 1 m below the surface. A total of 409 zooplankton samples were collected during the study, 46–91 samples from each of the 6 reservoirs. The sampled material of 20 L was filtered through a plankton net with a mesh size of 30 μm, preserved with Lugol's solution and fixed in a 4% formalin solution. Zooplankton were identified down to the lowest taxonomic level (with the exception of juvenile copepod stages) using a Zeiss AXIO Imager microscope according to the methods described by von Flössner^80^, Koste^81^, Streble and Krauter^82^, Ejsmont-Karabin et al.^68^, Rybak & Błędzki^64^ and Błędzki & Rybak^83^. In total, we identified 76 zooplankton taxa, including 22 Crustacea, 6 Protozoa and 48 Rotifera. Quantitative analyses included determination of zooplankton abundance (ind L^−1^) using a Sedgewick-Rafter counting chamber. Zooplankton biomass (mg L^−1^) was determined according to the methods proposed by Bottrell et al.^84^ and Ejsmont-Karabin^85^. Taxa with an abundance of less than 1% were removed from further analysis and modelling.

Physical and chemical data were collected simultaneously with the zooplankton, but in a central stand in the reservoir. Water temperature was additionally measured during the winter months (November–February) of two years. The sampling sites were located in the central part of the reservoir in each case. Water temperature (temp, °C) and dissolved oxygen (O2, mg L^−1^) were measured using the YSI 6600 V2 multiparameter water quality probe. A Secchi disc was used to measure transparency (SD, m). Water samples were collected for laboratory analyses of chlorophyll a concentration (µg L^−1^), total nitrogen (Ntot, mg L^−1^), ammonium nitrogen (NH4N mg L^−1^), nitrate nitrogen (NO3N, mg L^−1^), total phosphorus (Ptot, mg L^−1^) and phosphate phosphorus (PO4P, mg L^−1^). The total suspended solids concentration (SS, mg L^−1^) and the organic (SSorg, mg L^−1^) and inorganic (SSmin, mg L^−1^) fractions were determined. Hydrochemical analyses were carried out in accordance with APHA^86^ guidelines. The total number of samples from 6 lakes was 138 during the same periods as for the zooplankton samples.

### Assumption of modelling

Predicting changes in the biomass of zooplankton taxa under the influence of environmental changes requires a number of methodological prerequisites. First, we based our calculations on the interactions between the zooplankton species in the studied reservoirs resulting from the relationships between variations in their biomass. The effects of these interactions were described by modelling several combinations of a system of taxa in relation to each other with successive model boosts. For this purpose, the boosting technique of the eXtreme Gradient Boosting (XGBoost) algorithm was applied. Subsequently, the zooplankton taxa were treated as participants in a cooperative survival game using SHapley Additive exPlanations (SHAP) modelling. In the following chapters, the modelling procedure and the algorithms used are described in detail. A limitation of this approach is, of course, the lack of consideration of other biocenotic relationships of planktonic animals, such as phytoplankton grazing or predation by fish.

We created two classification databases: 1. zooplankton taxa (76 attributes and 409 observations), in which we assigned the biomass of taxa from three artificial lakes with lower winter temperatures, called Cold Lakes, to class "0" and the same taxa from three reservoirs with higher winter temperatures, called Warm Lakes, to class "1", and 2. physicochemical factors (11 attributes and 138 observations), with the same classification of the same thermal groups of lakes as above.

Our basic ecological assumption is to translate the significant difference in winter water temperatures of the Cold and Warm Lakes reservoir groups into characteristics of the biomass of zooplankton taxa in the studied months of the year. In the case of zooplankton biocenosis components, the stake in this game ("gain") is the extent of adaptation to thermal changes in the aquatic environment. The response of a particular taxon to increased water temperatures in winter may take the form of a reaction in that it tends to:

- increase biomass to varying degrees,

- decrease biomass to varying degrees,

- not respond to thermal water changes in winter.

To broaden the field of environmental interpretation of biomass changes in planktonic species, we modelled the response of a number of physicochemical factors to winter temperature increases in the waters of the reservoirs studied (XGBoost and SHAP). In the case of the physicochemical factors, we were concerned with the functioning of a system of these variables determined by the pursuit of a physical and chemical equilibrium in the water tone. The response of a particular physicochemical factor to winter heat anomalies can:

- increase its magnitude to varying degrees,

- decrease its magnitude to varying degrees,

- not respond to increased water temperature in winter.

### Data modelling scheme

To predict the changes in the biomass of zooplankton taxa under the influence of winter warming of the studied artificial lakes, a sequence diagram of data preparation for modelling and subsequent modelling was prepared. The original database of zooplankton taxa in Excel consisted of 409 biomass measurements of 76 taxa. The reference class "0", represented by biomass measurements in reservoirs with natural winter thermals (Cold Lakes), comprised 204 measurements and the predictive class "1" in reservoirs with higher winter thermals (Warm Lakes) comprised 205 measurements. The taxonomic classification of zooplankton into Crustacea, Rotifera and Protozoa was retained in the table. In contrast, the database of physicochemical factors was divided equally into the thermal classes Cold and Warm Lakes, with 69 cases each.

**Figure 5:**
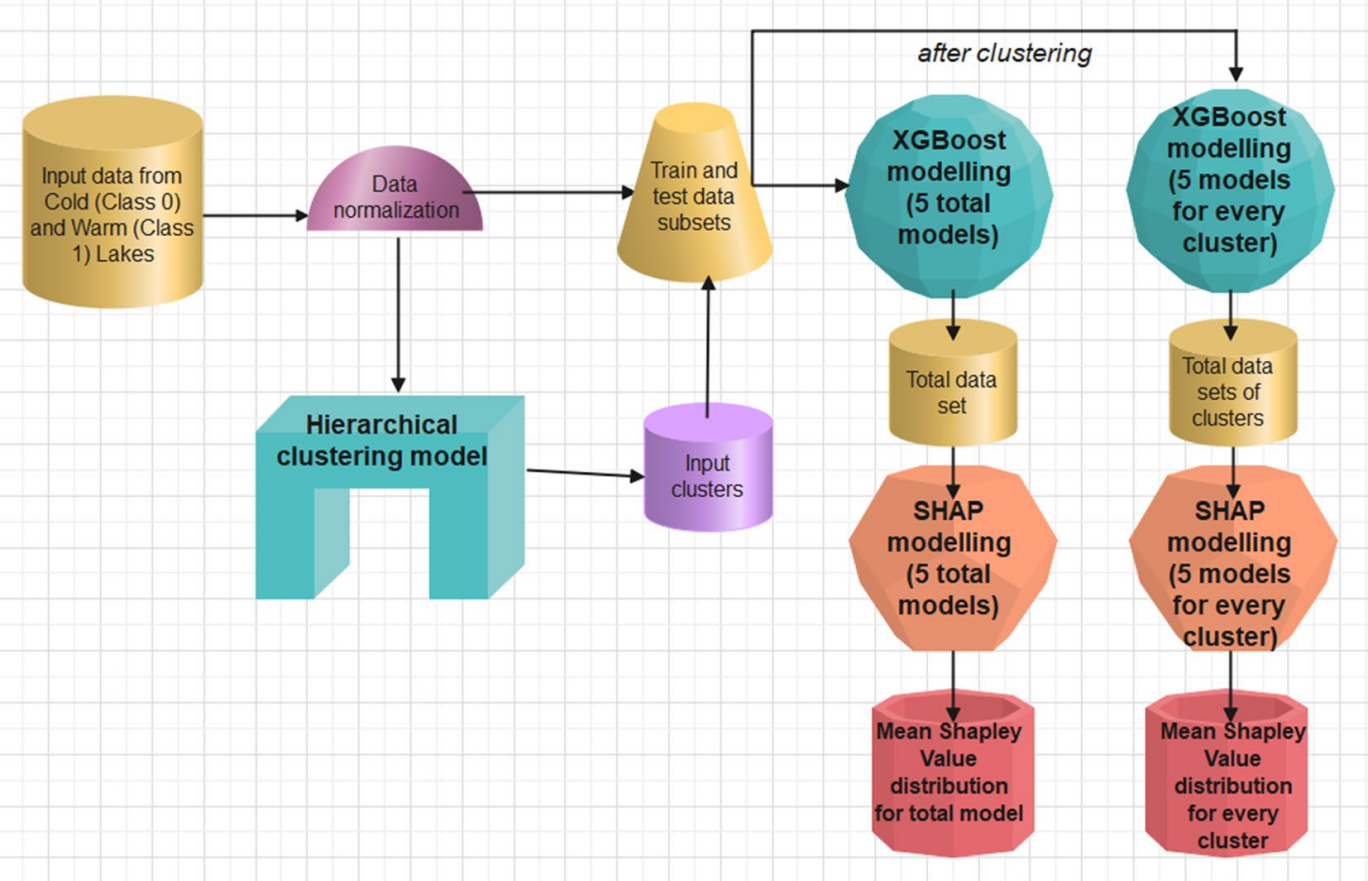
The scheme of the prediction modelling with alternative paths with and without clustering.

To transform the data table and adapt it for modelling with machine learning tools, we had to convert the Excel file into a text file with a csv extension and normalise the data with Min–Max Scaler. We carried out the next steps of data analysis and modelling in two variants. In the first variant, we used the entire database (76 features, 409 observations). After splitting the data into training and test subsets in a 70 to 30% ratio, we transferred them to modelling with the XGBoost algorithm. The training and test subsets were needed to calculate the accuracy of the model and check for overfitting. We then performed the modelling with the SHAP algorithm, again using the entire dataset. For the variant with zooplankton data, we subjected the entire dataset to the modelling procedure using the Hierarchical Clustering algorithm. After dividing 76 taxa into clusters, the results (predictions) were related to the individual separate clusters. For the XGBoost and SHAP models, 5 models were run randomly and the mean and standard deviation of the predictions of these five models were given as the final modelling results (Fig. 5). An analogous procedure was used for modelling the database of physicochemical factors of artificial lakes. Only the modelling variant involving a pathway with Hierarchical Clustering was dropped. All elements of data mining and modelling were carried out in the Python 3.8 language using the Jupiter Notebook programming environment.

### Hierarchical clustering

The use of the hierarchical clustering algorithm in this study was dictated by the fact that the zooplankton assemblage of a lake consists of 76 taxa with very different biomass, their distribution between observations, the abundance of the taxa and their role in the biocenosis. We assumed that building a single overall model based on interspecific interactions would not be sufficient to capture the responses of individual taxa to the thermal effect under study. We proposed to group the studied zooplankton community in analogy to the selection of operational taxonomic units (OUT) used in microbiological research^87^.

The grouping was based on the cophenetic distance, a measure of how similar two objects must be to each other to be classified in the same cluster. This distance is mathematically expressed by the cophenetic correlation^55^. This clustering method is widely used in biostatistics, especially in taxonomic analyses. This is consistent with the use of this method in this study, where the data between which the Euclidean distance is calculated are the biomasses of the zooplankton taxa. We used code from the Kaggle programming site created by Sgalella^88^.

### Gradient boosting modelling

The Extreme Gradient Boosting (XGBoost) algorithm is a popular and effective tree model-based learning technique in machine learning applications. The gradient boosting technique weights each weak classifier into a strong classifier and reduces the residuals of the previous models in the direction of the gradient to obtain a new model^89^. In general, this method mimics ecosystem processes by inhibiting the tendency to increase entropy^36^. XGBoost is the most effective tool among machine learning based boosting techniques^90^.

In our study, the Extreme Gradient Boosting tool was used as an introductory model for modelling SHAP to assess the prediction of changes in the biomass of zooplankton taxa as a result of warming winter reservoirs. The hyperparemeters of the model were as follows: n_estimators = 1000, max_depth = 10, learning_rate = 0.001. This algorithm was also used to predict the importance of physicochemical parameters of lakes for their classification into Cold and Warm Lakes. The argument for using XGBoost was also supported by the highest accuracy values calculated for a range of environmental parameters among several models tested in the analysis of their sensitivity to climate dynamics under shallow lagoon conditions^40^. We adopted a code from the Kaggle notebook 'Ensembles and Model Stacking'^91^.

### SHAP models

The SHAP algorithm^45^ is based on the concept of Shapley value as part of mathematical game theory and its branch describing cooperative games^44^. This modelling can be used to predict the local importance of variables^46^. If we consider the interactions between species in a biocoenosis as a game for resources and, more broadly, for adaptation and survival^48^, then modelling from SHAP can successfully serve as a tool to predict and determine trends in changes in the living components of an ecosystem under the influence of variability in environmental factors. An example of this is the prediction of the sensitivity of the biotic and abiotic components of the Vistula Lagoon ecosystem under the influence of changes in weather conditions from season to season^40^. The cited work provides a more comprehensive description of the adaptation of the Shapley value concept to the prediction of changes in biocenosis components under the influence of instability of the physical environment.

In the present study, the Shapley value was used to predict the trend of biomass changes of 76 zooplankton taxa during the growing season under the influence of winter warming of artificial reservoir water. To analyse the environmental context of the changes in the assemblage of these aquatic organisms, modelling from SHAP was also used to predict the changes in eleven physicochemical parameters. The changes in biological and physicochemical parameters can be positive or negative, and a parameter can also prove insensitive to these changes. Since the Shapley value plot for the traits in the model contains the individual position of these values for each observation, we applied the function ABS _SHAP, which synthetically indicates the predominant positive or negative change in a trait (taxon biomass or abiotic factor). We obtained the corresponding code from Github platform^92^. Diagrams showing the individual Shapley value distribution for each observation (biomass measurement) can be found in the Supplementary Information. The final output of the SHAP modelling for interpretation was the mean Shapley values from five random runs of the model. We used the model code from Medium Towards Data Science service^93^.

## Supplementary Information

Supplementary Information.

## Data Availability

The datasets generated during and analyzed during the current study are not publicly available due to rules established by the Project Funder but are available from the corresponding author on reasonable request.
