# Supplementary material for: Predicting the effects of winter water warming in artificial lakes on zooplankton and its environment using combined machine learning models
Source: Sci Rep. 2022 Sep 27;12:16145. doi: 10.1038/s41598-022-20604-x (PMC9515112; doi:10.1038/s41598-022-20604-x)
Supplement: Supplementary file 1 — Supplementary Information. [file 41598_2022_20604_MOESM1_ESM.docx]

**Supplementary Material**

**TITLE:**

**Predicting the effects of winter water warming in artificial lakes on zooplankton and its environment using combined machine learning models.**

*Kruk M., Goździejewska A., Artiemjew P.*

Table S1. Significant differences at p < 0.05 in an average biomass of the zooplankton taxa and their frequency (Freq) in the Cold and Warm Lakes during the study (n = 409). Significant differences have been marked in bold fond. The frames indicate in which lake type a higher taxa biomass occurred on average. The source of the Table: 96. Goździejewska, A.M. & Kruk M. Zooplankton network conditioned by turbidity gradient in small anthropogenic reservoirs. Sci. Rep. 12, 3938; https://doi.org/10.1038/s41598-022-08045-y (2022)..

| **Taxa** | **Cold Lakes** | | | **Warm lakes** | | | Kruskal - Wallis test | |
| --- | --- | --- | --- | --- | --- | --- | --- | --- |
|  | Biomass (µg/L) | | Freq | Biomass (µg/L) | | Freq |  |  |
|  | 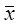 | ±SD | (%) | 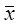 | ±SD | (%) | H | P |
| **Rotifera** |  |  |  |  |  |  |  |  |
| *Anuraeopsis fissa* | 0.036 | 0.157 | 9 | 0.000 | 0.000 | 0 | 2.3824 | 0.12271 |
| *Ascomorpha ovalis* | 16.98 | 51.09 | 55 | 5.447 | 19.059 | 32 | **24.4532** | **< 0.00001** |
| *Asplanchna priodonta* | 202.98 | 583.79 | 37 | 18.48 | 114.48 | 9 | **25.9268** | **< 0.00001** |
| *Brachionus angularis* | 1.24 | 4.80 | 19 | 0.063 | 0.362 | 6 | **5.6131** | **0.01783** |
| *Brachionus calyciflorus* | 3.19 | 18.64 | 8 | 0.011 | 0.154 | 0.5 | 1.9008 | 0.16799 |
| *Brachionus leydigii* | 0.390 | 3.572 | 2 | 0.514 | 3.788 | 5 | 0.2549 | 0.61365 |
| *Cephalodella gibboides* | 0 | 0 | 0 | 0.020 | 0.162 | 2 | 0.182 | 0.66963 |
| *Cephalodella* spp. | 0.041 | 0.195 | 8 | 0.140 | 0.466 | 29 | **13.0732** | **0.0003** |
| *Colurella colurus* | 0.032 | 0.185 | 7 | 0.041 | 0.112 | 21 | **5.4576** | **0.01948** |
| *Colurella uncinata* | 0.012 | 0.113 | 2.0 | 0.021 | 0.139 | 5 | 0.3478 | 0.55533 |
| *Conochilus unicornis* | 7.47 | 66.65 | 2 | 0.015 | 0.218 | 0.5 | 0.0668 | 0.79602 |
| *Dicranophorous* sp. | 0.065 | 0.617 | 2 | 0.040 | 0.415 | 1 | 0.0291 | 0.86449 |
| *Euchlanis dilatata* | 0.051 | 0.546 | 1 | 0.051 | 0.320 | 3 | 0.1767 | 0.67421 |
| *Euchlanis* spp. | 0.111 | 0.787 | 2 | 1.204 | 5.966 | 14 | **4.1582** | **0.04143** |
| *Filinia longiseta* | 2.09 | 11.63 | 14 | 0.140 | 0.672 | 9 | 0.9828 | 0.32152 |
| *Hexarthra mira* | 4.15 | 17.39 | 13 | 0.105 | 0.547 | 5 | 2.1654 | 0.14114 |
| *Keratella cochlearis* | 5.63 | 14.84 | 66 | 0.171 | 0.320 | 40 | **50.8869** | **< 0.00001** |
| *Keratella quadrata* | 6.47 | 24.58 | 46 | 0.689 | 3.788 | 24 | **23.2642** | **< 0.00001** |
| *Keratella tecta* | 11.26 | 52.01 | 45 | 1.655 | 9.907 | 30 | **15.1523** | **0.0001** |
| *Keratella testudo* | 0.092 | 0.807 | 5 | 0.073 | 0.437 | 7 | 0.1021 | 0.74929 |
| *Keratella ticinensis* | 0.053 | 0.475 | 3 | 0 | 0 | 0 | 0.3603 | 0.54834 |
| *Keratella valga* | 4.15 | 14.63 | 29 | 0.079 | 0.310 | 15 | **9.6980** | **0.00184** |
| *Lecane* spp. | 0.081 | 0.300 | 14 | 0.113 | 0.247 | 30 | **6.6802** | **0.00975** |
| *Lepadella ovalis* | 0.029 | 0.337 | 2 | 0.003 | 0.023 | 2 | 0.0266 | 0.87041 |
| *Lepadella* spp. | 0.036 | 0.177 | 6 | 0.020 | 0.075 | 11 | 0.5719 | 0.44949 |
| *Monommata maculata* | 0.051 | 0.351 | 3 | 0.082 | 0.314 | 9 | 1.3532 | 0.24472 |
| *Mytilina mucronata* | 0 | 0 | 0 | 0.016 | 0.117 | 2 | 0.1165 | 0.73286 |
| *Notholca acuminata* | 0.213 | 0.983 | 7 | 0.176 | 0.865 | 8 | 0.0021 | 0.9633 |
| *Notholca squamula* | 0.719 | 4.341 | 10 | 0.263 | 0.851 | 21 | **3.8848** | **0.04873** |
| *Polyarthra longiremis* | 141.8 | 281.5 | 91 | 16.71 | 145.90 | 40 | **180.4551** | **< 0.00001** |
| *Polyarthra vulgaris* | 10.84 | 28.02 | 33 | 0.002 | 0.023 | 0.5 | **31.2857** | **< 0.00001** |
| *Pompholyx complanata* | 0.027 | 0.156 | 4 | 0.014 | 0.121 | 3 | 0.0339 | 0.85398 |
| *Pompholyx sulcata* | 0.797 | 3.626 | 14 | 0.005 | 0.038 | 2 | **4.4268** | **0.03538** |
| *Proales* sp. | 0.233 | 1.766 | 6 | 0.237 | 0.791 | 14 | 1.735 | 0.18777 |
| *Scaridium longicaudum* | 0 | 0 | 0 | 0.010 | 0.060 | 3 | 0.3568 | 0.5503 |
| *Synchaeta* spp. | 67.61 | 185.44 | 47 | 12.90 | 73.98 | 22 | **25.1783** | **< 0.00001** |
| *Testudinella patina* | 0.016 | 0.232 | 0.5 | 0.097 | 0.546 | 7 | 1.2185 | 0.26965 |
| *Trichocerca insignis* | 0 | 0 | 0 | 0.060 | 0.333 | 5 | 0.881 | 0.34792 |
| *Trichocerca intermedia* | 0.000 | 0.006 | 0.5 | 0.003 | 0.017 | 5 | 0.7231 | 0.39511 |
| *Trichocerca musculus* | 0.003 | 0.047 | 0.5 | 0.005 | 0.032 | 3 | 0.1795 | 0.67176 |
| *Trichocerca myersi* | 0 | 0 | 0 | 0.011 | 0.081 | 3 | 0.2621 | 0.60866 |
| *Trichocerca porcellus* | 0 | 0 | 0 | 0.010 | 0.079 | 2 | 0.182 | 0.66963 |
| *Trichocerca pusilla* | 2.13 | 11.55 | 16 | 0.002 | 0.013 | 2 | **5.6807** | **0.01715** |
| *Trichocerca similis* | 0.002 | 0.027 | 0.5 | 0.018 | 0.201 | 2 | 0.0653 | 0.79828 |
| *Trichocerca tenuior* | 0 | 0 | 0 | 0.033 | 0.135 | 9 | 2.6286 | 0.10496 |
| *Trichocerca tigris* | 0 | 0 | 0 | 0.080 | 0.729 | 7 | 1.6383 | 0.20056 |
| *Trichotria pocillum* | 0.017 | 0.131 | 2 | 0.018 | 0.092 | 4 | 0.1708 | 0.67941 |
| *Trichotria tetractis* | 0 | 0 | 0 | 0.018 | 0.094 | 4 | 0.466 | 0.49483 |
| **Cladocera** |  |  |  |  |  |  |  |  |
| *Acroperus harpae* | 0 | 0 | 0 | 0.098 | 0.852 | 2 | 0.0655 | 0.79796 |
| *Alona affinis* | 0 | 0 | 0 | 0.293 | 2.956 | 2 | 0.0655 | 0.79796 |
| *Alona quadrangularis* | 0.123 | 1.259 | 1 | 1.066 | 5.399 | 8 | 1.4122 | 0.23469 |
| *Bosmina longirostris* | 120.6 | 337.7 | 34 | 14.38 | 63.59 | 22 | **7.6354** | **0.00572** |
| *Ceriodaphnia quadrangula* | 0.074 | 1.050 | 0.5 | 0.244 | 2.086 | 2 | 0.0291 | 0.86449 |
| *Chydorus sphaericus* | 0.289 | 3.227 | 2 | 0.312 | 1.786 | 4 | 0.2557 | 0.61306 |
| *Daphnia cucullata* | 6112 | 11585 | 76 | 32.56 | 88.80 | 24 | **137.9877** | **<0 .00001** |
| *Graptoleberis testudinaria* | 0 | 0 | 0 | 0.366 | 3.129 | 2 | 0.0655 | 0.79796 |
| *Leptodora kindtii* | 25.98 | 127.1 | 7 | 0 | 0 | 0 | 1.4412 | 0.22995 |
| **Copepoda** |  |  |  |  |  |  |  |  |
| *Acanthocyclops robustus* | 3.55 | 29.82 | 3 | 0 | 0 | 0 | 0.2647 | 0.60691 |
| *Cryptocyclops bicolor* | 12.88 | 50.81 | 12 | 0.146 | 2.10 | 0.5 | **3.9211** | **0.04768** |
| *Cyclops strenuus* | 14.33 | 44.62 | 14 | 0 | 0 | 0 | **5.7647** | **0.01635** |
| *Cyclops vicinus* | 20.51 | 67.89 | 12 | 0 | 0 | 0 | **4.5956** | **0.03205** |
| *Diacyclops crassicaudis* | 0 | 0 | 0 | 0.732 | 5.30 | 2 | 0.182 | 0.66963 |
| *Eucyclops speratus* | 0.147 | 2.100 | 0.5 | 0.878 | 7.22 | 2 | 0.0655 | 0.79796 |
| *Eudiaptomus graciloides* | 29.22 | 200.69 | 4 | 0 | 0 | 0 | 0.5956 | 0.44027 |
| *Harpacticoida* | 0.404 | 3.428 | 2 | 0.659 | 4.15 | 4 | 0.176 | 0.67482 |
| copepodites | 275.2 | 459.7 | 87 | 6.37 | 17.25 | 29 | **183.4908** | **< 0.00001** |
| *Metacyclops minutus* | 0.980 | 8.126 | 2 | 0 | 0 | 0 | 2.4671 | 0.1176 |
| *Microcyclops varicans* | 10.88 | 37.66 | 10 | 0.488 | 5.75 | 1 | 2.4199 | 0.1198 |
| nauplii | 58.74 | 83.36 | 85 | 1.244 | 2.33 | 60 | **147.7965** | **< 0.00001** |
| *Thermocyclops crassus* | 44.78 | 140.05 | 17 | 0.146 | 2.10 | 0.5 | **10.17** | **0.00143** |
| **Protozoa** |  |  |  |  |  |  |  |  |
| *Arcella discoides* | 0.005 | 0.026 | 4 | 0.039 | 0.112 | 29 | **18.0503** | **0.00002** |
| *Centropyxis aculeata* | 0.042 | 0.505 | 5 | 0.008 | 0.029 | 12 | 1.2869 | 0.25663 |
| *Codonella cratera* | 16.20 | 48.74 | 62 | 0.018 | 0.082 | 14 | **92.9679** | **< 0.00001** |
| *Difflugia acuminata* | 0.002 | 0.02 | 2 | 0.001 | 0.010 | 1 | 0 | 0.99666 |
| *Difflugia lobostoma* | 3.689 | 11.457 | 26 | 0.030 | 0.100 | 15 | **6.072** | **0.01373** |
| *Difflugia pyriformis* | 0 | 0 | 0 | 0.002 | 0.016 | 2 | 0.1165 | 0.73286 |

Table S2. Significant differences at p < 0.05 in the average value of physicochemical parameters in the Cold and Warm Lakes during 2 years in the study period. The higher value of a parameter in a lake class was marked in bold. The Kruskal-Wallis test was used for n=138 observations for all parameters, except for water temperature in winter (November - February), marked with *, where n=24.

| **Parameter** | **Units** | **Cold Lakes** | | **Warm lakes** | | Kruskal - Wallis test | |  |
| --- | --- | --- | --- | --- | --- | --- | --- | --- |
|  |  |  |  |  |  |  |  |  |
|  |  | 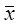 | ±SD | 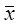 | ±SD | H | p |  |
| Water temperature (temp) | C^o^ | 15.01 | 6.05 | 16.86 | 3.39 | 1.3317 | 0.24850 |  |
| Winter water temperature* | C^o^ | 5.64 | 1.87 | **12.25** | **3.04** | 14.5200 | 0.00014 |  |
| Oxygen concentration (O2) | mg/l | **9.10** | **1.29** | 8.50 | 1.27 | 6.4195 | 0.01129 |  |
| Secchi Disc visibility (SD) | m | 0.71 | 0.20 | **0.97** | **0.45** | 10.0103 | 0.00156 |  |
| Chlorophyll a | µg/l | **6.40** | **6.18** | 2.76 | 3.58 | 33.3663 | <0.0001 |  |
| Phosphate Posphorus (PO4P) | mg/l | 0.023 | 0.016 | 0.023 | 0.013 | 0.2440 | 0.62133 |  |
| Total Phosphorus (Ptot) | mg/l | 0.112 | 0.109 | 0.105 | 0.063 | 0.0002 | 0.98981 |  |
| Ammonium Nitrogen (NH4H) | mg/l | 0.072 | 0.053 | 0.092 | 0.073 | 2.4224 | 0.11961 |  |
| Nitrate Nitrogen (NO3N) | mg/l | **0.152** | **0.066** | 0.131 | 0.064 | 7.5789 | 0.00591 |  |
| Total Nitrogen (Ntot) | mg/l | **0.307** | **0.124** | 0.247 | 0.105 | 9.7426 | 0.00180 |  |
| Suspended Solids mineral (SSmin) | mg/l | **3.06** | **3.31** | 1.71 | 1.70 | 16.2103 | 0.00006 |  |
| Suspended Solids organic (SSorg) | mg/l | 4.30 | 5.00 | 3.39 | 3.31 | 0.2503 | 0.61683 |  |

Individual 5 plots of cluster 1 zooplankton taxa SHAP model


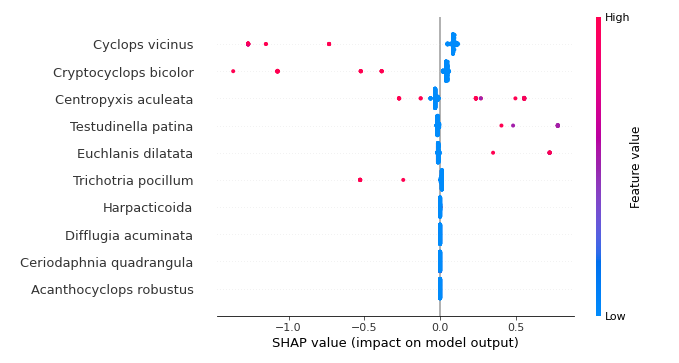

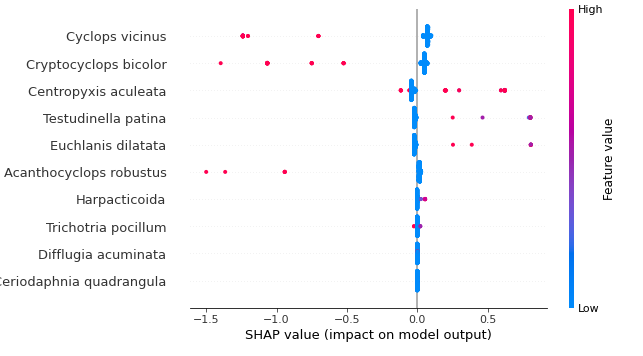


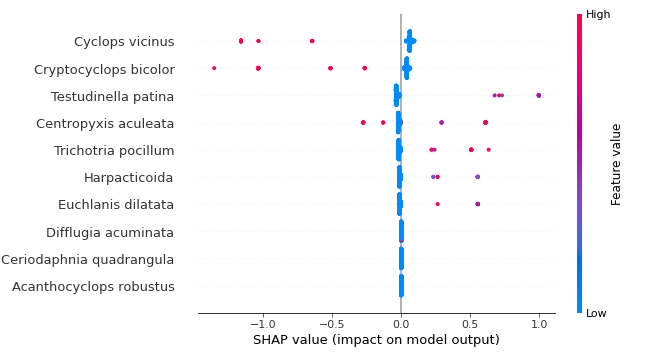

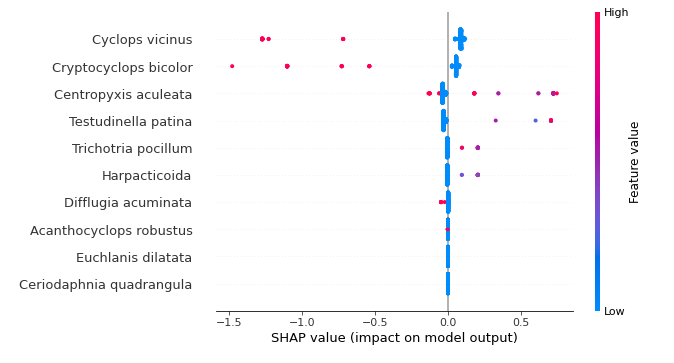


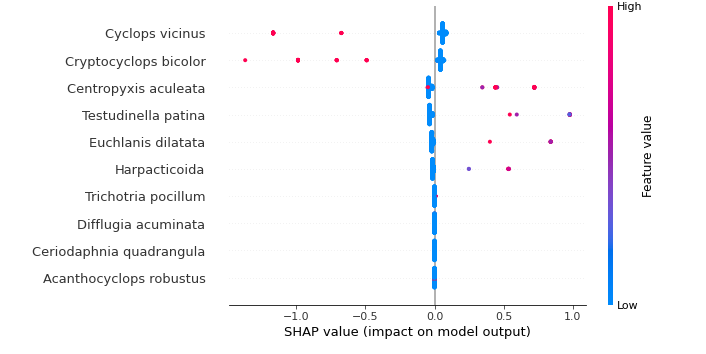

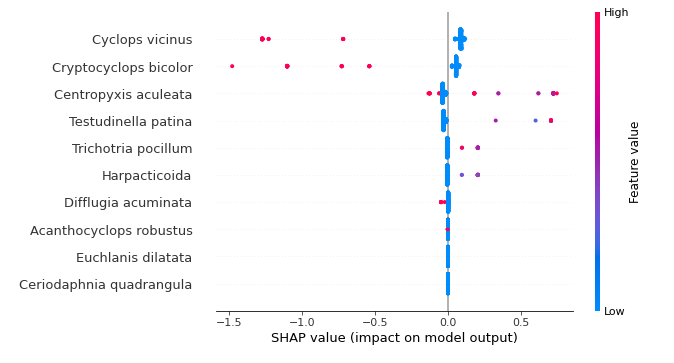


Individual 5 plots of cluster 2 zooplankton taxa SHAP model


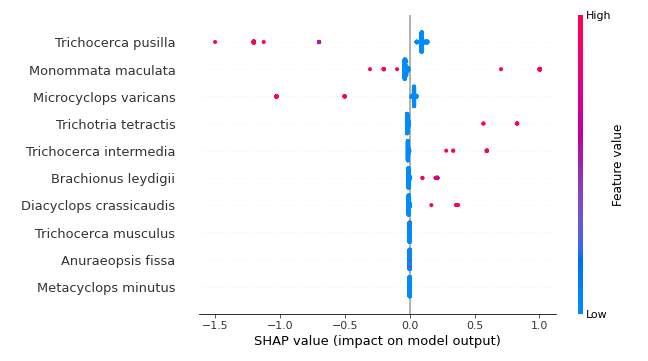

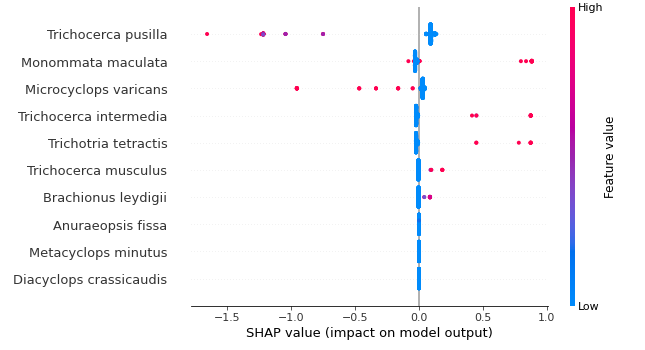


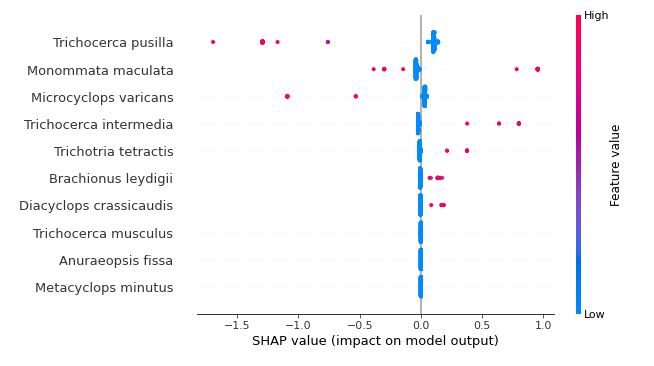

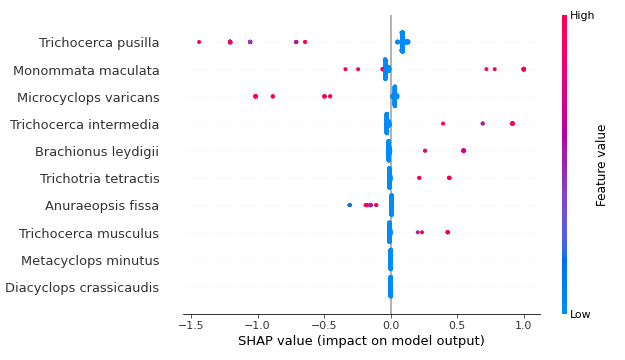


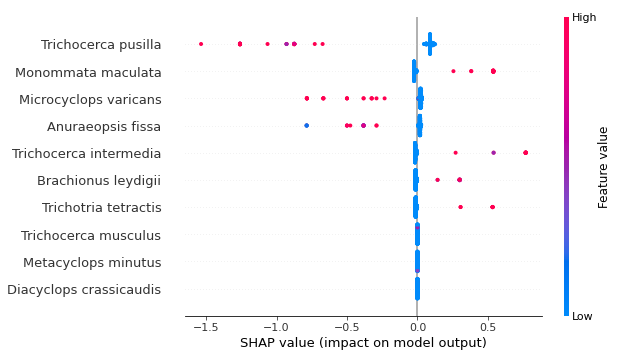


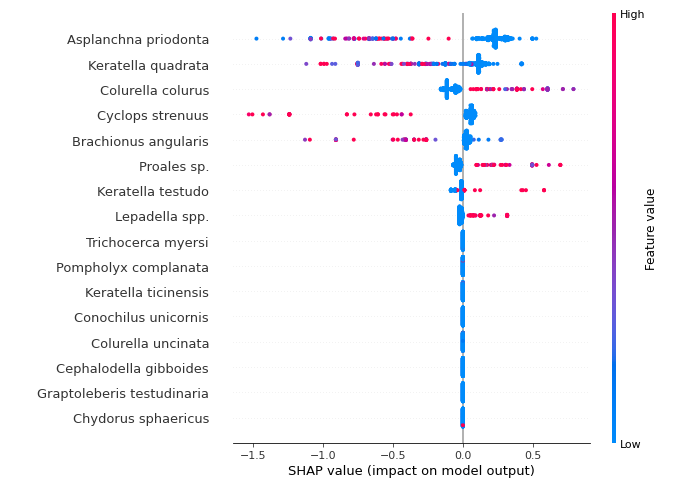

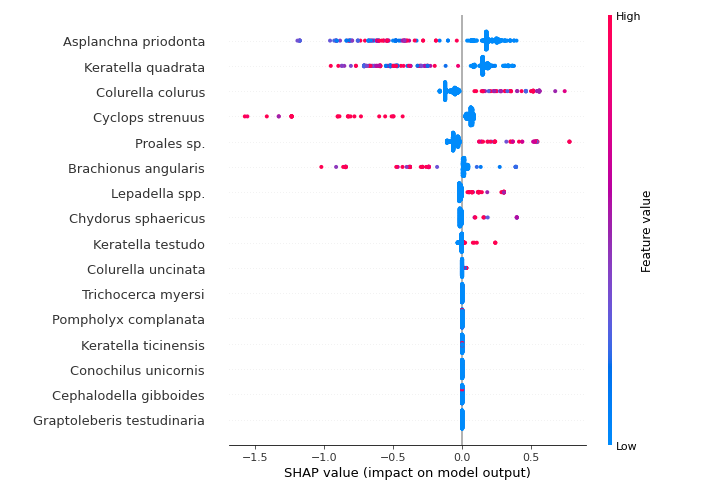
Individual 5 plots of cluster 3 zooplankton taxa SHAP model


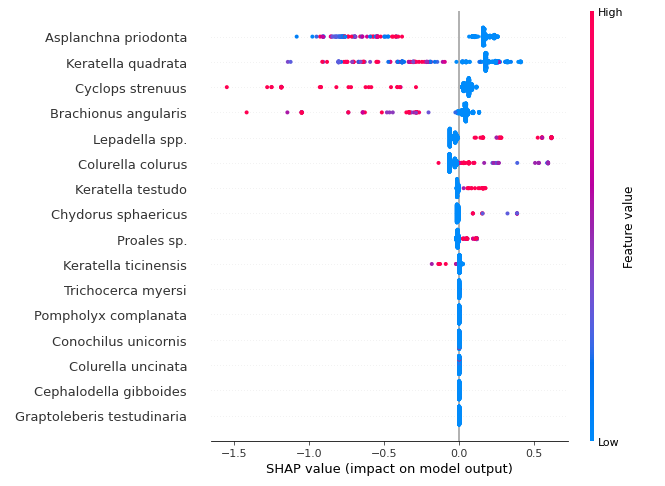

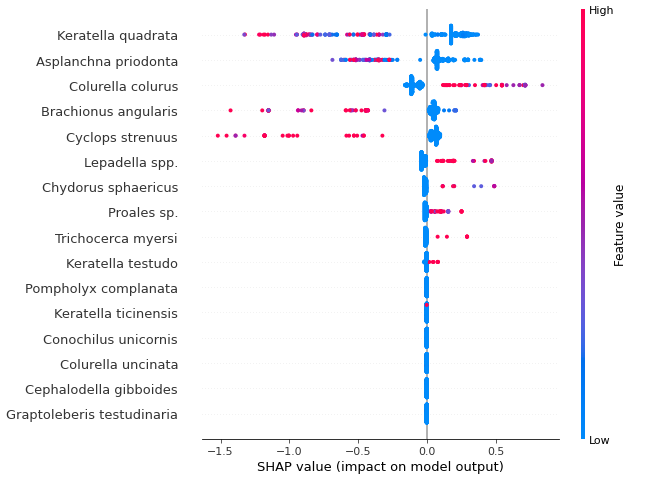


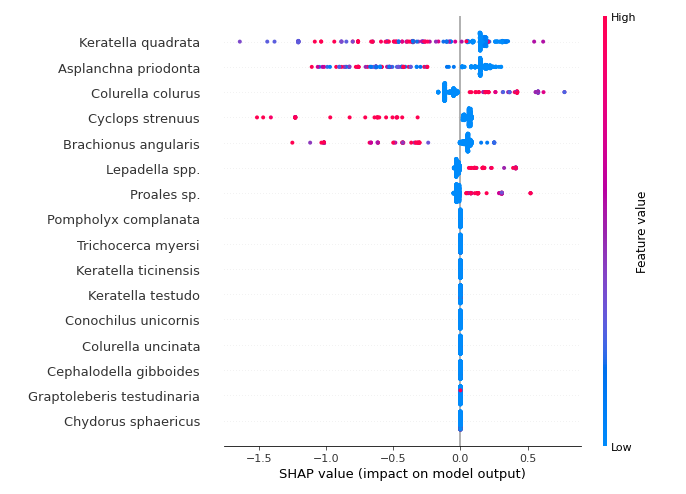


Individual 5 plots of cluster 4 zooplankton taxa SHAP model


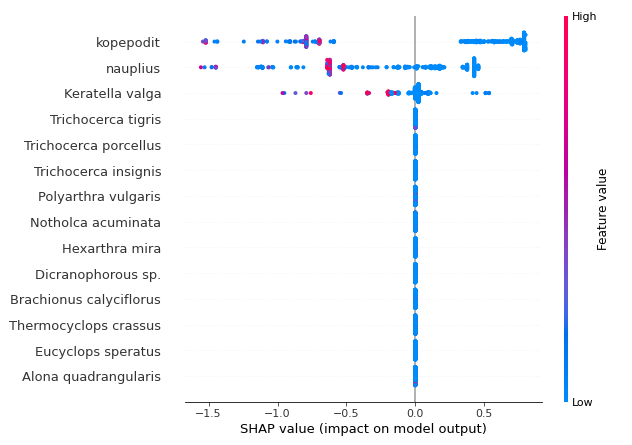

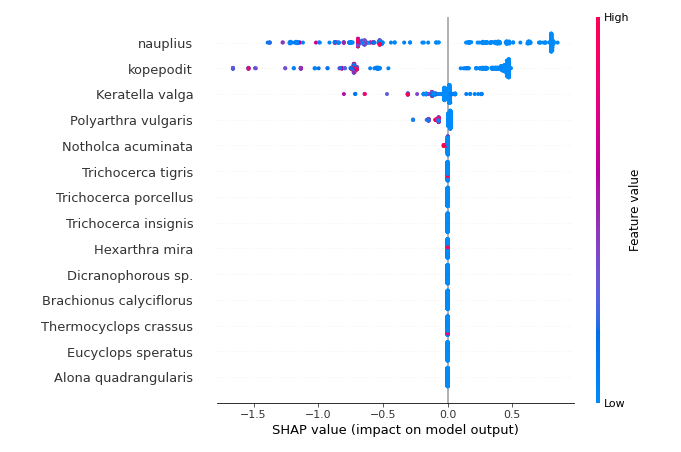


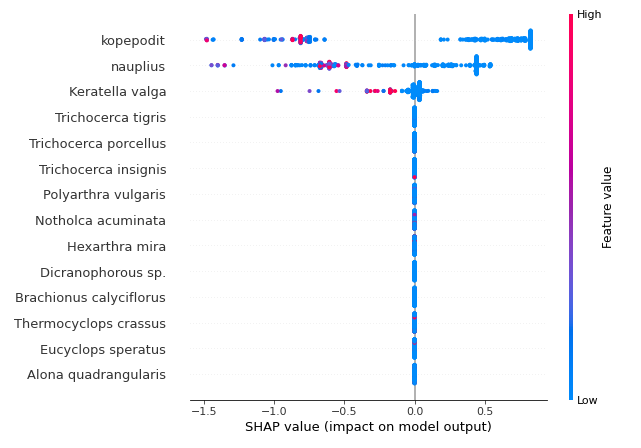

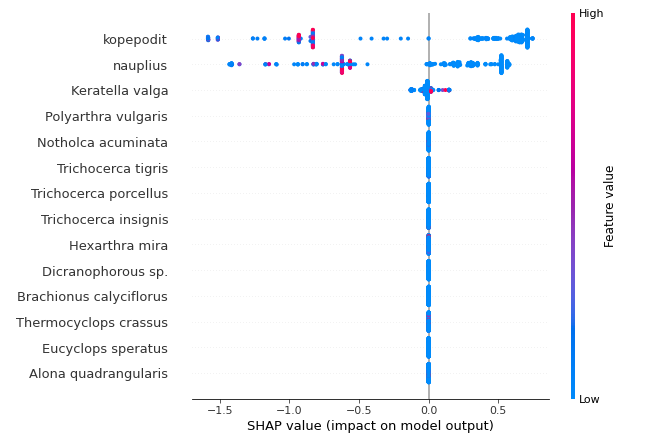


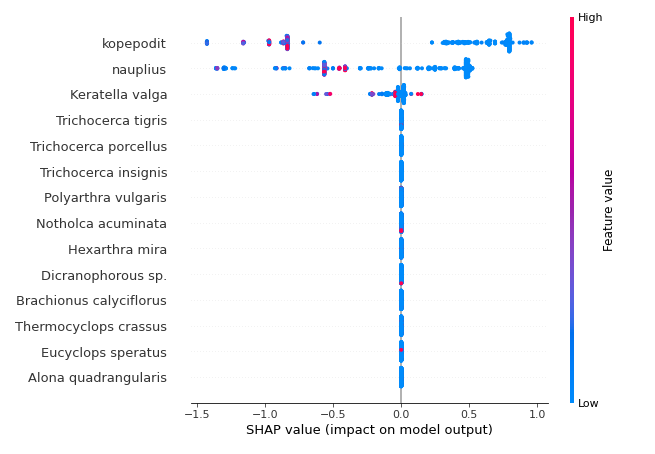


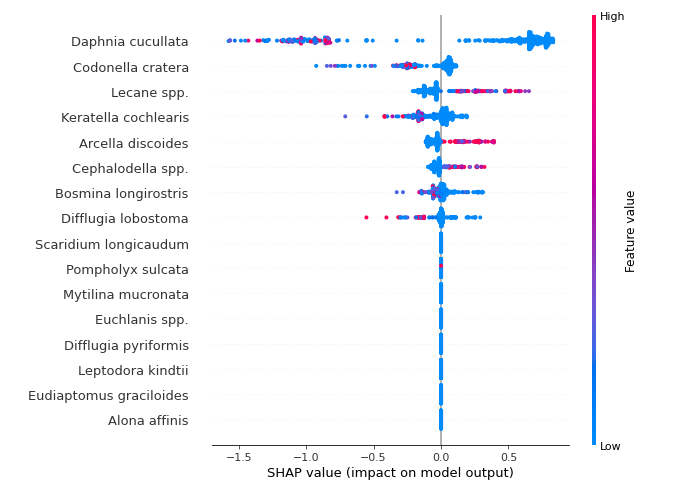

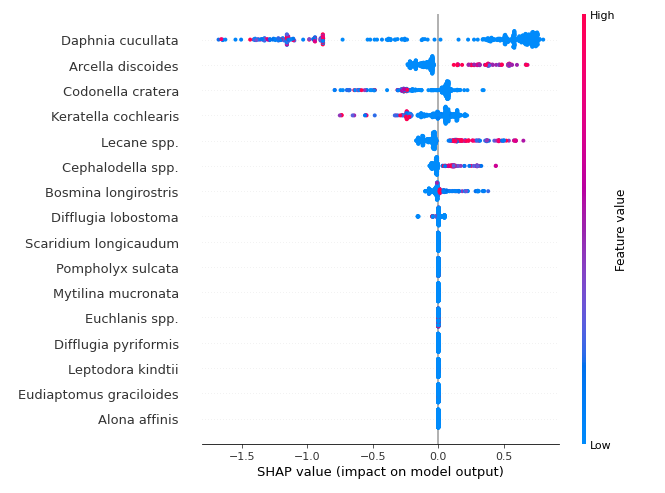
Individual 5 plots of cluster 5 zooplankton taxa SHAP model


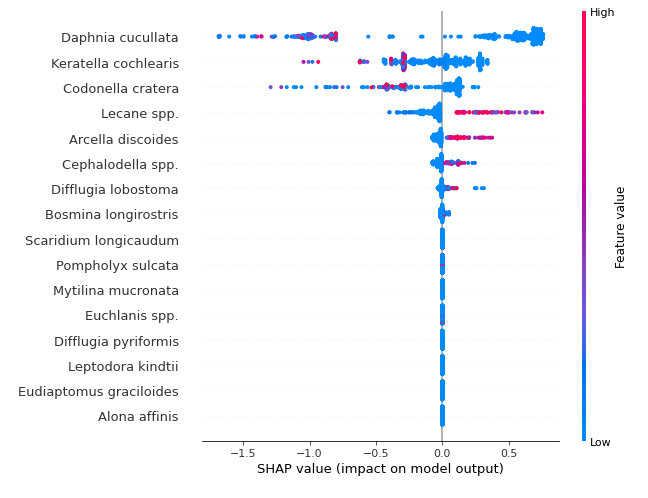


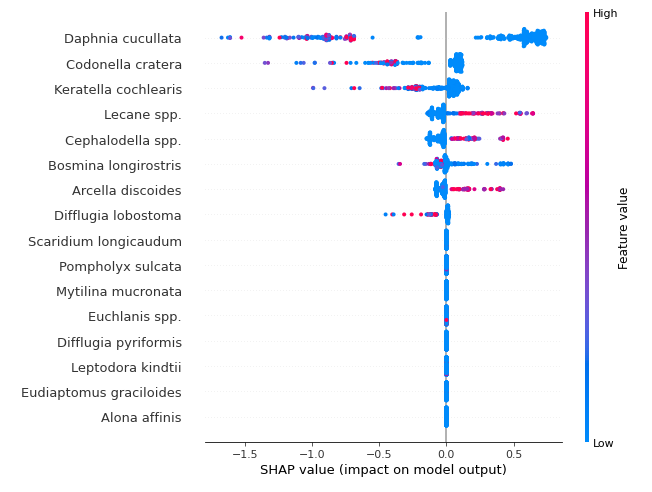


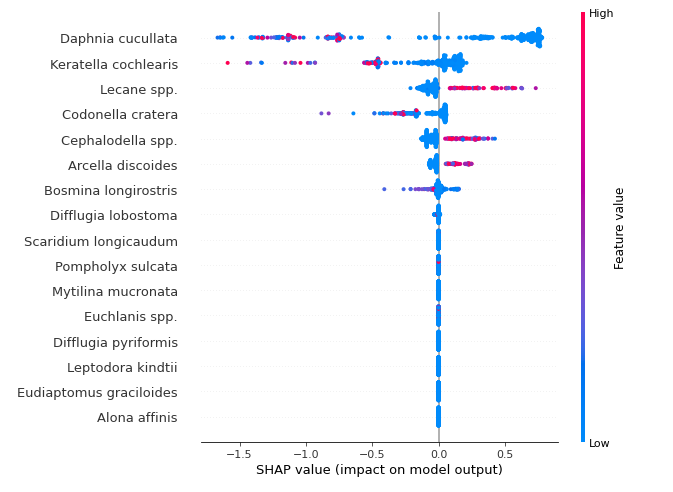


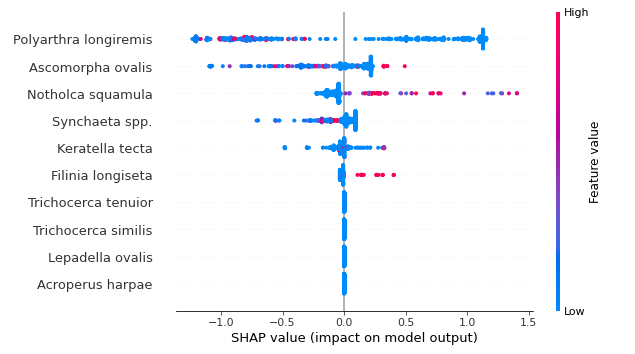
Individual 5 plots of cluster 6 zooplankton taxa SHAP model


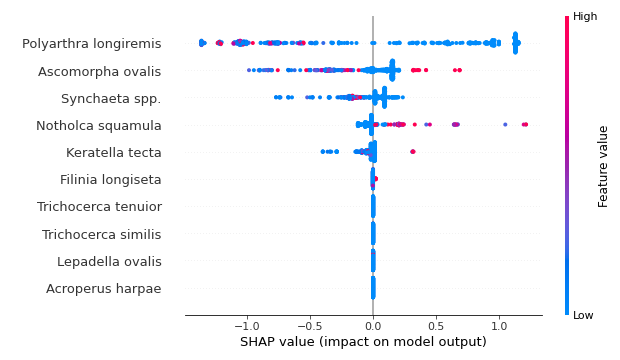


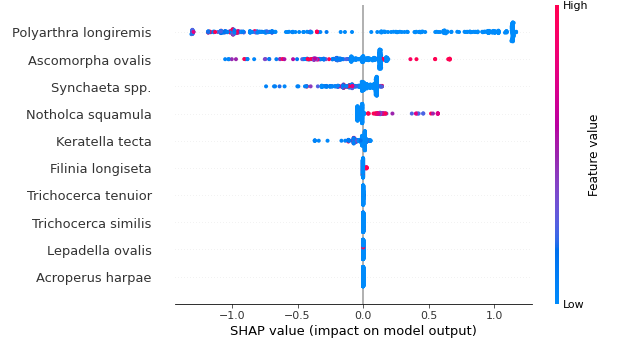


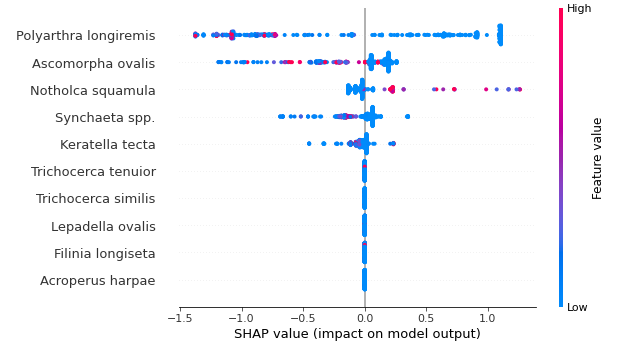


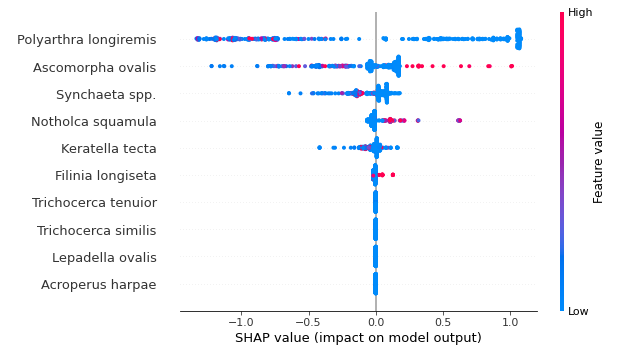


Fig. S1. Individual plots of six zooplankton taxa SHAP models of clusters (referred to Fig. 3 in the main text).


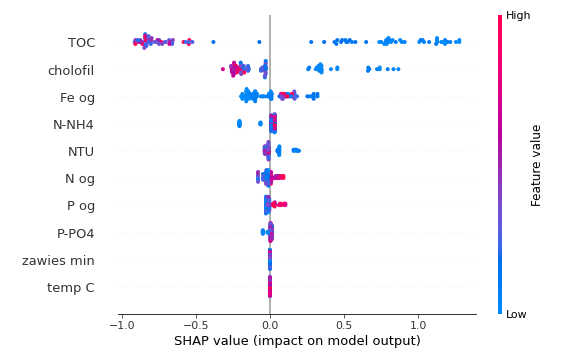
Individual 5 plots of physicochemical SHAP model


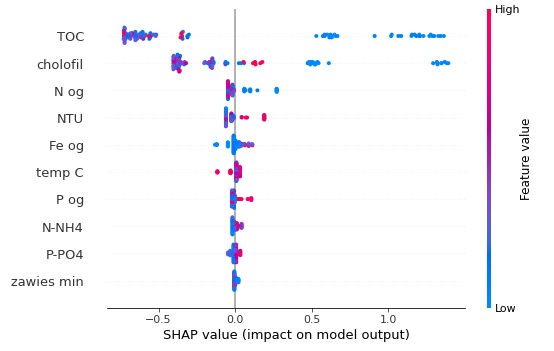


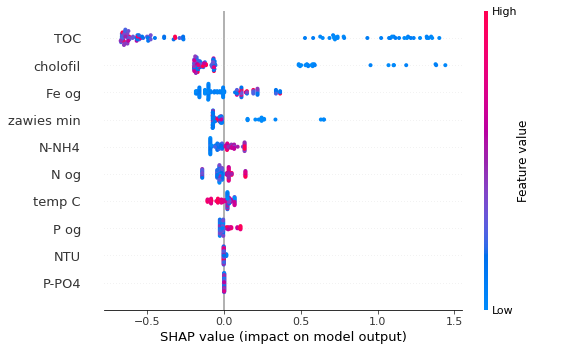

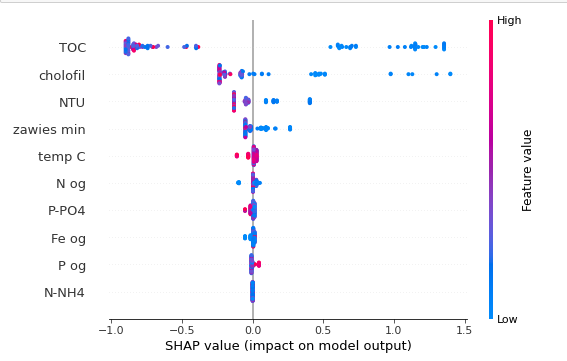


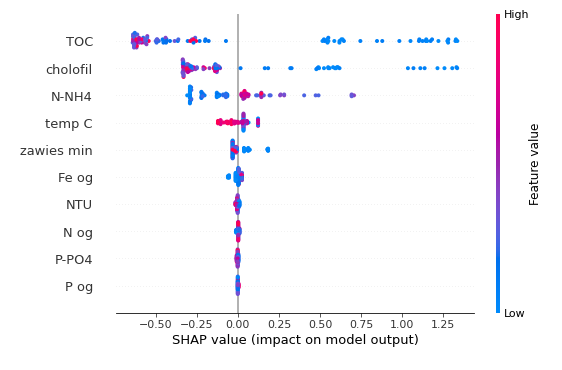


Fig. S2. Individual 5 plots of physicochemical SHAP model (referred to Fig. 4 in the

main text).


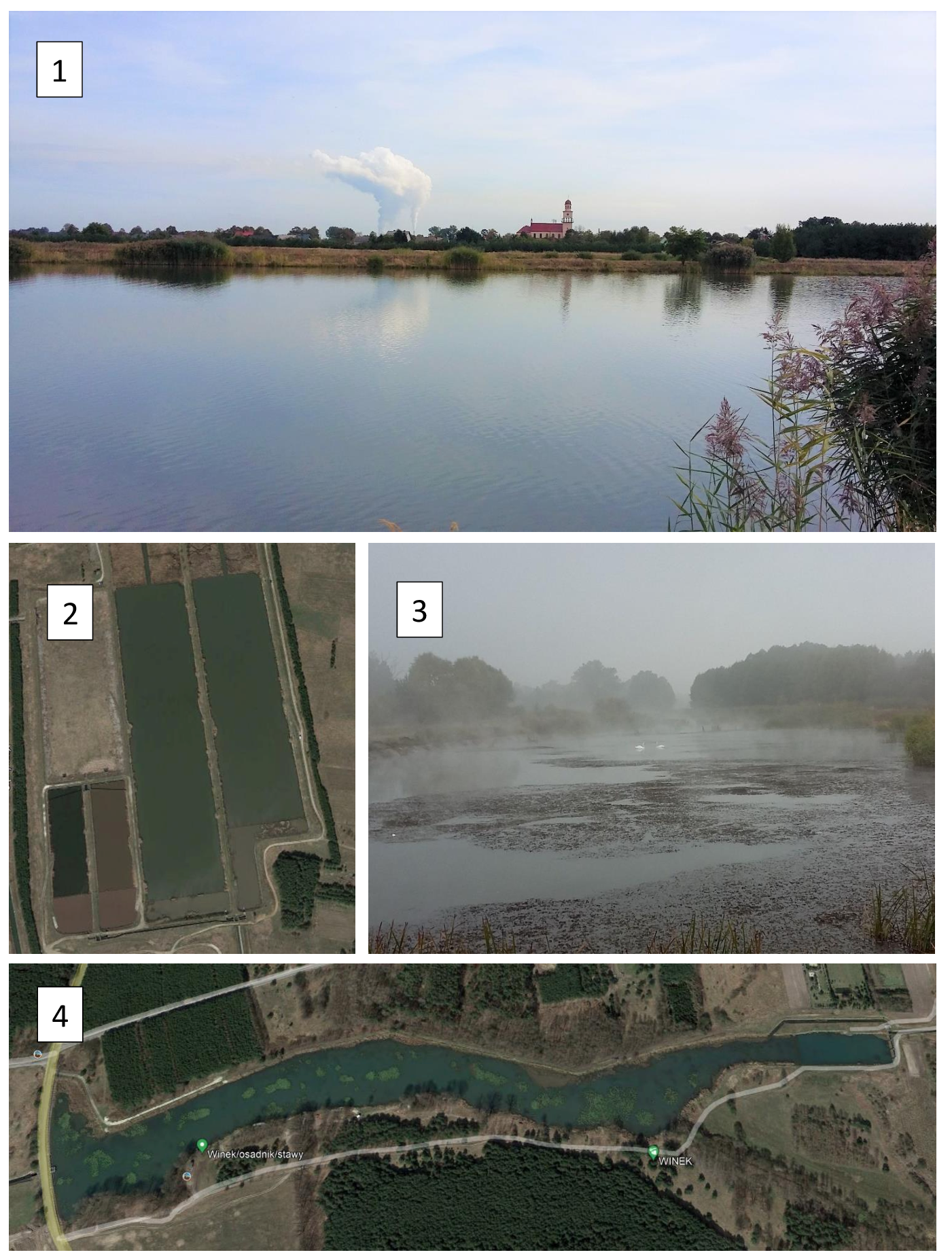


Fig. S3. Field and satellite photos of 1. and 2. Cold Lakes CH1 and CH2, 3. and 4. Warm Lake WI. Photos 1. and 3 by A.M. Goździejewska. Photos 2. and 4. Google Earth Pro images (Attribution information: Image ©2022 Maxar Technologies, Image ©2022 CNES/Airbus).
